# Vero cells gain renal tubule markers in low-calcium and magnesium chemically defined media

**DOI:** 10.1038/s41598-022-10221-z

**Published:** 2022-04-13

**Authors:** Megan Logan, Karsten Rinas, Brendan McConkey, Marc G. Aucoin

**Affiliations:** 1 Department of Chemical Engineering, University of Waterloo, Waterloo, ON N2L 3G1 Canada; 2 Department of Biology, University of Waterloo, Waterloo, ON N2L 3G1 Canada

**Keywords:** Chemical engineering, Biochemistry, Cell biology, Cell adhesion, Cell growth

## Abstract

In this study, a chemically defined, animal component-free media was developed to promote Vero growth in suspension. Key media compounds were screened using Plackett–Burman styled experiments to create a media formulation to support suspension growth. Vero cells remained viable in suspension, but their growth rate was extremely low, conversely, other cell types such as CHO-K1, MDCK and HEK293T were able to grow in single cell suspension in the same media. To investigate the slow growth of Vero cells, RNA-seq analysis was conducted. Vero cells were cultured in three different conditions: adherently in serum-containing medium, adherently in in-house medium, and in suspension in low calcium and magnesium in-house medium. This study illustrates that adherent cells maintain similar gene expression, while the suspension phenotype tends to overexpress genes related to renal tubules.

## Introduction

Vero cells have been used for vaccine production because they are permissible to a plethora of viruses and are approved in over 60 countries for biomanufacturing^1^. Unfortunately, one of the major limitations of Vero cells is that they are almost exclusively cultured adherently. For large scale manufacturing, cells can be grown adherently in roller bottles, cell stacks, or on microcarriers; however, their ability to be cultured to a high cell density is limited by the available surface area. Large gains in productivity for protein and virus production have been made by adapting cells to suspension^2^. This allowed researchers to increase the maximum cell density and to eliminate the cost of microcarriers^3^, not to mention reducing the complexity of the overall process. To improve virus production for vaccines, cells grown in suspension culture would ease scale up, remove the cost associated with the microcarriers or roller bottles, and has the potential to maximize productivity per volume. For example, Rourou et al*.*, were able to show that suspension Vero cells could produce more rabies virus (3 × 10^7^ FFU) compared to adherent cells grown on Cytodex 1 microcarriers (1 × 10^7^ FFU)^4^. Ideally, Vero cells could be grown in single cell suspension in bioreactors with animal component free (ACF) media, without reducing product quantity or quality.

Previous work by Litwin in 1992 has demonstrated that Vero cells can be grown in suspension as aggregates in a serum-free medium^5^. Since then, other cell lines such as CHO, MDCK, BHK-21, HEK and HeLa cells have been successfully grown as single cell suspension, but researchers were not able to culture Vero cells in suspension until 2019. Two groups published the ability to grow Vero cells around the same time. Rourou et al*.*, used an in-house medium to culture Vero cells in suspension and were able to replicate rabies virus^4^. Shen et al*.,* were also able to culture Vero cells, using a separately developed in-house medium and produced vesicular stomatitis virus (VSV) in a 3 L bioreactor^6^. While this is a major triumph for vaccine manufacturing, the cells grew at approximately half the rate of serum-containing medium, and both media contained undefined plant hydrolysates. Ideally, Vero cells could be grown in suspension using a chemically defined media that is also animal component-free. Chemically defined medium (CDM) is formulated such that all the components and their respective concentrations are known. CDM formulations may contain proteins and serum-derived components, and therefore are not guaranteed to be protein-free or animal-component-free. The knowledge of every component and their concentration allows for better control during the biomanufacturing process. It also has the benefit of simplifying the downstream purification process since it reduces the complexity of removing unknown components^7^.

Designing a chemically defined medium is no easy task. Although basic formulations have been published, most media development remain as trade secrets. The number of components that can be added to a formulation, and their concentration ranges, make the design space almost impossible to tackle. The use of design of experiments (DoE) can help somewhat. Ideally, one would want to use full factorial DoEs, as these types of design can shed light on interactions between medium components; however, the number of experimental conditions can become impossible to run. One option is to use a Plackett–Burman DoE. With the Plackett–Burman design, one can test the greatest number of factors in the least number of experimental runs by accounting for only main factor effects. In other words, this style of experiment will not lead to understanding whether the main factor effects change because of changing levels of other factors. This is the limitation of the design; however, it does provide a rational for maintaining or removing compounds in the development of a medium.

Although the medium in which a cell is cultured will impact transcriptional regulation, understanding what genes are being transcribed can also inform the formulation of the medium. RNA-Seq and proteomic work has been done to identify changes between adherent and suspension cells for cell lines such as CHO-K1, HeLa, MDCK, and HEK293^8–11^. The aim of this work was to examine the transcriptional changes of Vero cells adapted to a medium that can sustain Vero cells in a single-cell suspension phenotype. First, the development of the medium is described, followed by a transcriptomics analysis of the cells maintained in this medium compared to those maintained in DMEM/F12 + 10% FBS and a version of the CDM with higher levels of Mg and Ca. Insights from this work provide a novel reason for why it has been difficult to culture Vero cells in suspension.

## Results and discussion

### Plackett–Burman media screening for chemically defined medium development

**Figure 1:**
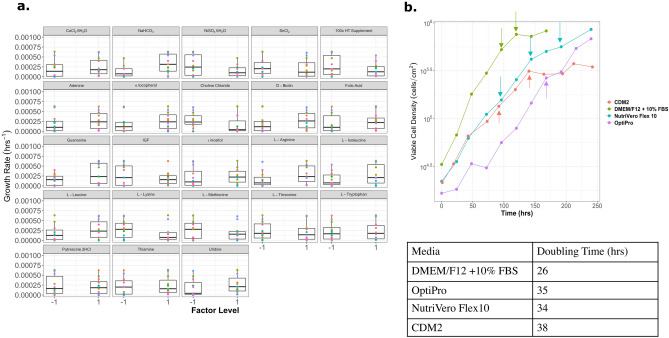
(**a**) Box plots that show the effect of each media component for a Plackett–Burman experiment with 23 factors in 24 runs. This experiment used a basal media with low calcium and magnesium to encourage suspension growth. The growth rate was use as the dependent variable to compare the influence of each media component. (**b**) The adherent chemically defined media (CDM) formulation was compared to commercial media. The arrows indicate when media was exchanged. CDM2, CDM3, and CDM4 were formulated based on the results from Plackett–Burman experiments 4, 6 and 7, respectively.

Four Plackett–Burman styled experiments were conducted to rapidly screen media components to support suspension growth in Vero cells starting from a DMEM/F12 base. The major carbon source was glucose, which was increased from 17.5 to 25 mM, and the l-alanyl-l-glutamine concentration was increased to 4 mM. In total, 62 compounds were tested over 96 formulations in the four different Plackett–Burman experiments (see Supplementary Table S1). From this, a chemically defined adherent and suspension medium formulations (CDM) were made for Vero cells. The medium formulations were improved iteratively with each subsequent Plackett–Burman experiment, and the base formulation for the next experiment was designed from the previous set of results. An example of the results from a Plackett–Burman experiment designed for suspension media is illustrated in Fig. 1a, where the effect of each component on growth rate is shown. Through this experiment, the effect of purines and pyrimidines (adenine, guanosine, uridine, and HT supplement) can be seen having overall a positive effect on growth rate, with the exception of the HT supplement which was supplemented at too high of a concentration for the highest factor level. Another category of compounds that improved the growth rate was metals. In addition to metals such as calcium, magnesium, iron, copper and zinc, various trace metals were added to the medium formulation. Specifically cobalt, maganese, molybdenum, silicate, selenite, nickel, tin, and vanadium, which are normally excluded from basal media, but can be found in serum, were critical for cell viability and growth^12^. Trace metals are important cofactors in various enzymes, and these metals can be found in deionized water, but not in ultra pure water. One of the drawbacks of adding metals to protein-free media, is that metals are pro-oxidants and can lead to the oxidation of media if antioxidants or metal chelators are not present. Antioxidants such as α-tocopherol (Vitamin E), ascorbic acid (Vitamin C), glutathione, citric acid, and pyruvate were added to the medium. Of these compounds, all of them were beneficial at high concentrations, with the exception of glutathione, which had a detrimental effect on cell growth at concentrations greater than 3.3 µM (Supplementary Fig. S3). All the experiments and the concentration of each factor level can be found in the Supplementary Figs. S1–S4.

Growth factors, polyamines, vitamins and steroids were investigated to increase the growth rate. Surprisingly, not all growth factors had a positive effect on the growth rate of Vero cells, insulin-like growth factor (IGF) had no poistive effect, while recombinant epidermal growth factor (rEGF) was required for growth (Fig. 1 and Supplementary Fig. S1). More recent media formulations developed for Vero cells have used rEGF^13,14^ rather than fibroblast growth factor (FGF). Cinatl et al*.*, found that Vero cells could grow well on polyvinyl formal flasks in a protein free media that contained progesterone^15^. It is likely that rEGF could be replaced by a combination of steroids to create a protein-free medium for Vero cells.

The adherent chemically defined medium contained 10 ng/mL of rEGF as the only protein present in the final formulation and achieved a doubling time of 32 h, compared to OptiPRO SFM (35 h) and NutriVero Flex10 (34 h) (Fig. 1b). Epidermal growth factor (EGF) is commonly added to chemically defined cell culture media because the EGF receptor (EGFR, also known as ErbB1) is expressed on almost all cell types. Plant hydrolysates have been known to mimic growth factor-like effects for many different cell types at low cost and is animal component-free (ACF), which makes them a good alternative to supplementing with individual growth factors^16–18^. OptiPRO SFM was chosen as a comparison because it is commonly used in industry as a medium to produce vaccines using Vero cells. This medium is animal component-free but it does contain a very low protein concentration of plant hydrolysates (≤ 10 μg/mL)^19^. NutriVero Flex10 is another Vero cell medium that is commercially available, and unlike OptiPro, it is chemically defined, as well as ACF, making it a better comparison to the medium developed in this paper.

Overall, none of the ACF media formulations (NutriVero Flex 10, OptiPRO SFM, or CDM2) grew as fast as serum-containing DMEM/F12 with 10% FBS. Therefore, one could infer that there are still some components missing from ACF media such as cell signaling molecules that are in low concentrations, or growth factors that are missing from serum-free media. A study conducted by Desai et al*.*, tracked the growth factors that were produced by Vero cells^20,21^. They found that Vero cells excreted platelet derived growth factor (PDGF), interleukin 6 (IL-6) and leukemia inhibitory factor (LIF), but were unable to detect EGF or active TGF-β. Interestingly, Guo et al., were only able to find EGFR on Vero cells when they conducted a proteomic analysis of the membrane proteins^22^. This may have been due to the limited annotation of the Vero genome and surface proteins, or that the EGFR in Vero cells can interact with many different ligands. Nevertheless, rEGF did stimulate proliferation of Vero cells adherently in the chemically defined medium, but the literature suggests that other growth factors could be used in addition, or to replace rEGF.

### Suspension medium development

**Figure 2:**
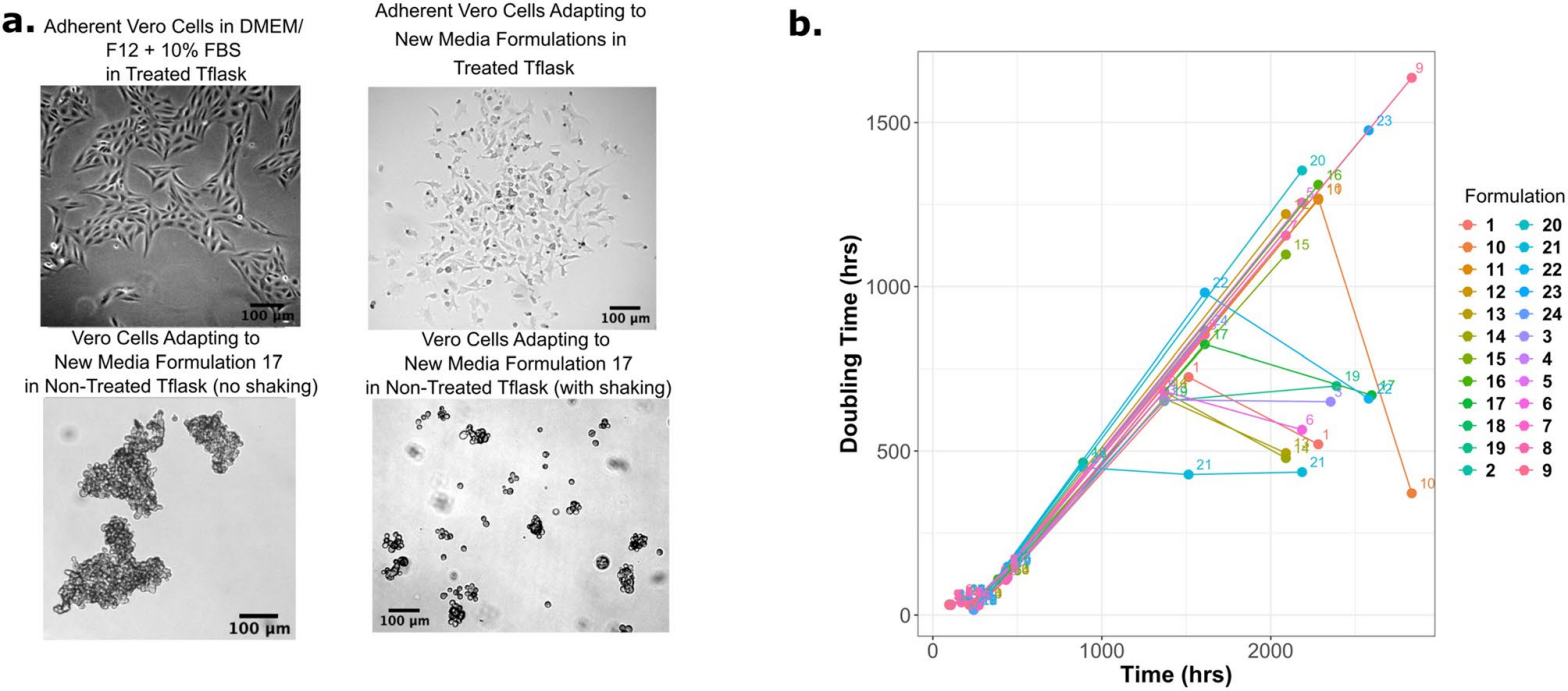
(**a**) Brightfield images of Vero cells as they adapt to low-calcium and magnesium media over the course of 180 days. Vero cells grown in DMEM/F12 + 10% FBS is used as a reference for normal morphology, compared to the cells growing in the chemically defined media (top right image). Vero cells were unable to adhere to non-treated T-flasks in the chemically define media, and with the addition of shaking (40 rpm, bottom right image) single cells and small aggregates were achieved. (**b**) The doubling time of Vero cells increased dramatically as they were adapted to the low-calcium and magnesium media over the course of 118 days. The shortest doubling time at the end of the experiment was approximately 20 days (500 h).

Cells were slowly adapted from DMEM/F12 + 10% FBS medium in adherent culture to the new medium formulations, and as the amount of calcium and magnesium decreased, the cell growth rate was also observed to decrease dramatically. At the lowest concentration range of 0.1 mM calcium, Vero cells were still able to adhere to the tissue-culture treated Tflasks; therefore, cells were transferred to non-treated Tflasks. Tissue culture treated Tflasks are commonly coated with polylysine, but Tflasks can also be coated with collagen, laminin or Matrigel to encourage cell attachment^23,24^. Through subsequent passaging in non-treated Tflasks, Vero cells detached and formed large aggregates (Fig. 2a). After 90 days of culturing in non-treated Tflasks, the cells were moved onto a shaker at 40 rpm in an effort to break up the cell aggregates. Only one formulation supported viable single cells (Formula 17).

Sodium bicarbonate (NaHCO_3_) had a large effect on the doubling time, which can be seen in Fig. 2b. There are two major groups in Fig. 2b, one group has a large doubling time (> 1000 h), and one with a shorter doubling time (~ 500 h). The faster growing group contained 2.2 g/L of sodium bicarbonate, while the slower group had 1.2 g/L. For 5% CO_2_ incubators, the concentration of NaHCO_3_ should be between 1.2 and 2.2 g/L, and 3.7 g/L for 10% CO_2_ incubators^25^. For our medium formulation, 2.2 g/L of sodium bicarbonate provided extra buffering. Even with the improvements seen from adding more NaHCO_3_, the doubling time of these cells was very lengthy (doubling time of 20 days), therefore further medium formulations focused on decreasing the doubling time.

One of the major differences between adherent media and suspension media is the concentration of calcium and magnesium. These two ions are important for cell adhesion proteins such as cadherins, which require a certain concentration (> 55 μM) of calcium to maintain the correct rigid conformation to remain active^26^. Calcium is also involved in many cellular processes besides cell adhesion^27^, such as cell signaling^28^, enzyme activity, and apoptosis^29^. Healthy cells maintain a large concentration gradient between the cytosol (0.1 μM) and extracellular space (1–2 mM)^27^. Magnesium is the second (after potassium) most abundant cation inside the cell and ranges from 17 to 20 mM in most mammalian cells^30^. Most of the magnesium is bound to various cellular structures, and only about 0.8–1.2 mM is free Mg^2+^^30^. It is essential for the most basic functions in the cell, for example, for ATP to be biologically active. Low Mg^2+^ concentration has also been linked to accelerated differentiation of bone-marrow-derived mesenchymal stem cells (MSCs) into osteoclasts through increased reactive oxygen species (ROS) generation^31^. While for adipose-derived MSCs the 10 × reduction (1 mM Mg, versus 0.1 mM Mg) of Mg in reprogramming medium caused the cells to have increased expression of genes associated with MSCs (*gata-4*, *nkx-2.5*, *hgf*, *kdr*, *nerog*, *nanog*)^32^. It is most likely that the reduction of these ions in the suspension medium (Formula 17) had detrimental effects on cell signaling, DNA replication, RNA transcription, and enzymatic activity. Potentially, the low magnesium levels could be causing transcriptional differences in the suspension cells that are causing them to differentiate. Subsequently, Formula 17 was modified to have the same concentration of calcium and magnesium ions as DMEM/F12 for comparison purposes, and was called CDM2. Formula 17 was thus renamed ‘CDM2 low calcium/low magnesium’ henceforth.

### Growth with other cell lines

**Figure 3:**
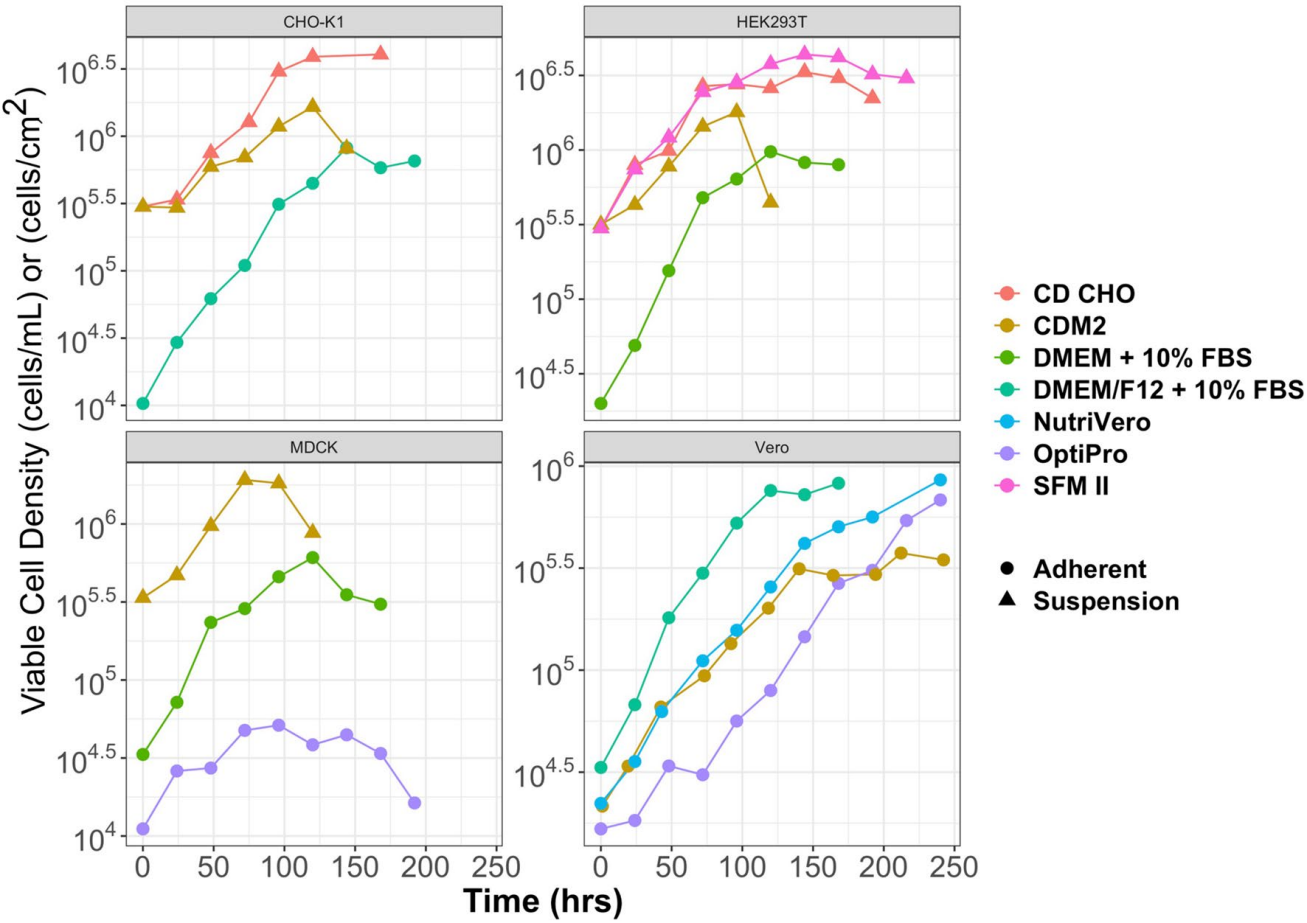
The chemically defined media that was formulated from the series of Plackett–Burman experiments was modified to include 1 mM Ca^2+^ and 2 mM Mg^2+^ (called CDM2) to support a higher growth rate in Vero cells. CHO-K1, HEK293T and MDCK cells were slowly adapted to CDM2 from serum-containing basal media. All three cell types were able to grow in suspension (triangle) when they were fully adapted to CDM2 and were cultured in shaker flasks. Vero cells remained adherent for all media formulations. Suspension (filled triangle), adherent (open circle).

To investigate if CDM2 could support growth of other cell lines, MDCK, CHO-K1 and HEK293T cells were sequentially adapted to CDM2 from serum-containing basal medium using static Tflasks. HEK293T and MDCK cells were adapted over 2 weeks, while CHO-K1 cells took 3.5 weeks to adapt to CDM2. Given that CDM2 contains the same amount of calcium and magnesium as basal DMEM/F12, this indicated that cells could still grow in suspension with higher levels of the divalent cations. Figure 3 demonstrates how the cells grew in CDM2 compared to commercial media, and serum-containing basal media, along with the maximum cell densities achieved when culturing the cells in suspension. For CHO-K1, HEK293T and MCDK cells grown in CDM2, cells started to lose their viability after approximately 4 days. After day 3, a significant color change in the medium could be seen indicating that the media was becoming acidic. Given that this medium was developed for adherent Vero cells, the ability to grow 3 other cell lines in suspension was a surprising result that led to the question of what is different about Vero cells that prevents them from growing well in suspension.

### RNA-seq experiment to cellular expression changes

To identify the reasons for the reduced growth of the Vero cells in suspension in a defined medium, a transcriptomic analysis was performed to identify expressional changes. This data helped to identify metabolic processes and transcription factors that could lead to a decreased doubling time in the chemically defined medium. The RNA-seq dataset comprised three groups of cells; (1) Vero cells grown adherently in DMEM/F12 + 10% FBS, (2) Vero cells grown adherently in CDM2 with added calcium and magnesium, or (3) Vero cells grown in suspension in CDM2. Cells grown adherently in DMEM/F12 medium (Control, or ‘Con’) and cells grown adherently in CDM2 with additional calcium and magnesium (Adherent, or ‘Adh’) were sequenced and compared to cells grown in suspension in CDM2 (Suspension, or ‘Sus’). A summary of the alignment statistics is provided in Supplementary Table S2. Over 96% of the reads for each sample were uniquely aligned to the *Chlorocebus sabaeus* (*C. sabaeus*) genome. After filtering the identified genes for an expression of at least 1 CPM in four samples, 11,135 genes were considered as expressed for further analysis (Supplementary Table S3). Due to the limited information about cellular pathways in *C. sabaeus*, the Ensembl database was used to identify *H. sapiens* homologs for the expressed genes. This allowed a more detailed gene set enrichment analysis. The complete results of the differentially expressed gene analysis are in Supplementary Tables S4–S7.

The variability within the dataset was analyzed with: (1) a principal component analysis (PCA) of the 500 most variable genes; and (2) a Pearson correlation of 3122 known, expressed housekeeping genes (Supplementary Fig. S5). The Pearson correlation of housekeeping genes controlled for outliers with unanticipated changes in housekeeping gene expression. The PCA showed a clear separation between all three conditions with Adh samples being grouped between Con and Sus samples as predicted.

### Changes in regulation of proliferation and apoptosis

**Figure 4:**
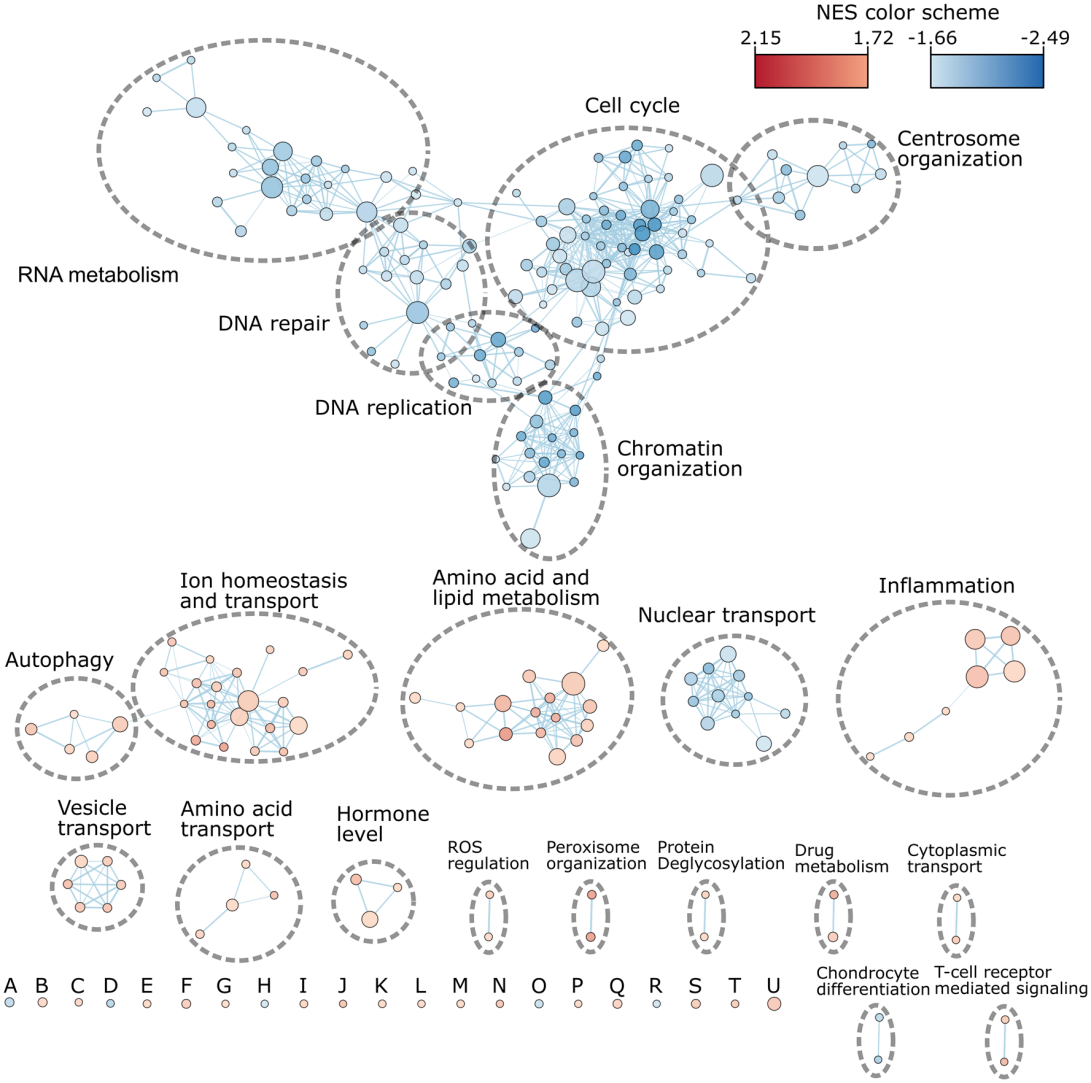
Enrichment map for significant downregulated GO-terms in biological processes for the comparison of Sus_CDM2 against Adh_CDM2 (threshold at FDR of 0.05). The size of the node is proportional to the number of identified genes annotated to the GO-term. The thickness of edges represents the level of overlap between the GO-terms. A: DNA templated transcription termination, B: Aerobic respiration, C: Response to increased oxygen levels, D: Protein peptidyl prolyl isomerization, E: Cofactor transport, F: positive regulation of inflammatory response, G: Cellular response to fluid shear stress, H: Regulation of ubiquitin protein ligase activity, I: Establishment of endothelial barrier, J: Hyperosmotic response, K: Regulation of protein oligomerization, L: Excitatory synapse assembly, M: Regulation of calcineurin mediated signaling, N: Modified amino acid transport, O: Cell migration involved in sprouting angiogenesis, P: Low density lipoprotein receptor particle metabolic process, Q: Endosome organization, R: Pyrimidine nucleotide triphosphate biosynthetic process, S: Water soluble vitamin metabolic process, T: Collagen catabolic process, U: Drug metabolic process. NES: Normalized enrichment score; The complete gene set list with group association is shown in Supplementary Table S8.

**Figure 5:**
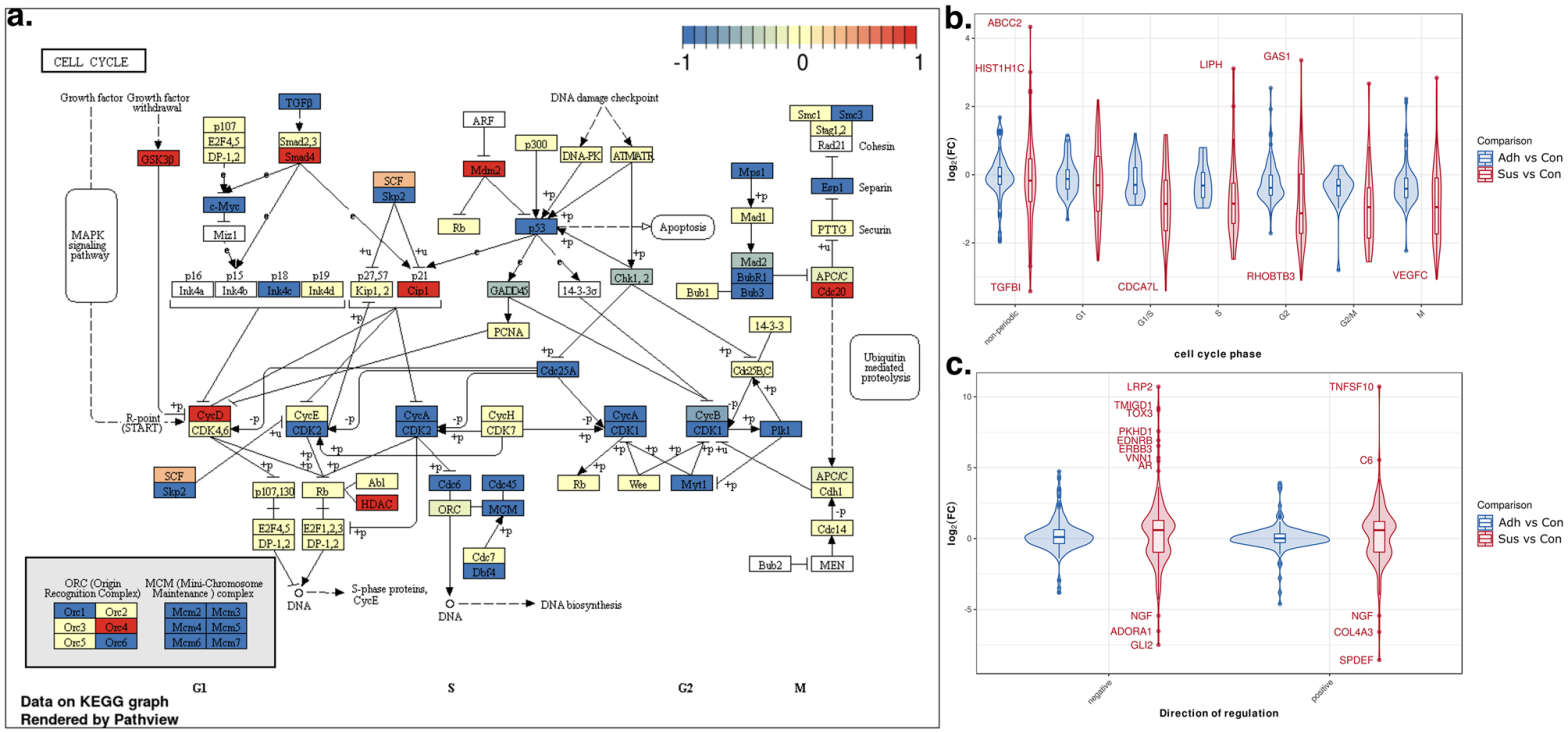
Expression regulation of cell cycle related genes. (**a**) KEGG cell cycle showing differentially regulated genes for the comparison of Sus_CDM2 against Adh_CDM2 rendered with Pathview. The color code determines the direction of significantly differentially regulated genes (FDR < 0.001) according to the legend at the top of the figure. A white background of a text box means that the gene was not identified as expressed. (**b**) LFC for the top 500 cell cycle related genes from Cyclebase 3.0 grouped to their peak expression phase. Non-periodic: no known expressional changes during the cell cycle, G1: Gap phase 1 of the cell cycle, G1/S: Gap 1 to synthesis transition phase, S: synthesis phase, G2: Gap phase 2, G2/M: Gap2 to mitosis transition phase, M: mitosis phase. (**c**) LFC for genes linked to positive (positive) or negative (negative) regulation of apoptosis according to gene ontology.

GSEA analysis was used to identify the most consistent expression changes in gene sets. An enrichment map of the significant down-regulated GO-terms in biological processes with a stringent FDR threshold of 5% can be seen in Fig. 4 (Supplementary Table S8). Overall, 151 gene sets were significantly down-regulated, and 91 were identified as significantly up-regulated. Most down-regulated gene sets were related to cell cycle regulation, mitosis, DNA-replication and DNA organization which is consistent with the observation of the long doubling time of Vero in suspension (Fig. 5a)^33–35^. Suspensions cells had down-regulated genes from each part of cell cycle progression compared to adherently grown Vero cells in CDM2 (Fig. 5b). The gene *c-myc* is down-regulated in suspension Vero cells, and this gene has been associated with cell cycle progression, along with tumorogenesis^36^. The overexpression of this gene has been shown to allow quiescent cells to reenter the cell cycle and begin to proliferate^36^. Up-regulated genes were linked to inflammation, fluid shear stress, migration and endothelial barriers, indicating a stress response during cell adaption to suspension. Importantly, gene sets related to regulation of programmed cell death were not detected as either down- or up-regulated. Genes sets regulating apoptosis were not detected as differentially expressed by GSEA, as seen by the expression patterns of positive regulatory (GO:0043065) and negative regulatory (GO:0043066) gene sets related to apoptotic processes (Fig. 5c). Despite a wider log fold-change (LFC) range for pro-apoptotic genes and anti-apoptotic genes, the average LFC for both sets of genes is approximately zero for Adh samples, and only slightly positive for Sus samples.

The highest LFC for anti-apoptotic genes *lrp2*, *pkhd1* and *tmigd1* have been shown to specifically protect renal cells from apoptosis^37–39^. On the other hand, for the top up-regulated pro-apoptotic genes, *tnfsf10* has been proven to trigger apoptosis in renal cells. The expression of apoptogenic genes like *bax*, *bak1*, or *bad* were unaffected, whereas, anti-apoptic genes are either unchanged like *mcl1* or *bcl2l2* or increase like *bcl2*^40^. Overall, the pattern does not seem conclusive for the Sus samples to indicate a change in the rate of apoptosis due to the widespread expression changes of both pro- and anti-apoptosis regulating gene sets.

### Mitochondrial fatty acid metabolism

The fatty acid (FA) beta oxidation pathway is the main up-regulated gene set in the GSEA amino acid and lipid metabolism cluster (Fig. 4). The product of this pathway, acetyl-CoA, is further converted in the Krebs cycle for energy production. The expression changes of FA beta oxidation in Sus samples are presented in Supplementary Fig. S6. The heatmap in Fig. S6b illustrates the upregulation of 29 beta oxidation associated genes. The schematic diagram in Fig. S6a illustrates that for each step in fatty acid beta oxidation at least one gene is up-regulated. This upregulation of enzymes for each main step might relate to an increased energy consumption. One key regulator of fatty acid metabolism in renal cells is *tgfb1*^41^ (encodes for transforming growth factor-β1 protein), which is down-regulated in Sus cells whereas its receptor *tgfb1r* is up-regulated. The expression of the *tgfb1* gene inhibits beta oxidation and promotes dedifferentiation, along with the epithelial-to-mesenchymal transition^42^. Differentiated tubular epithelial cells use FA beta oxidation as their main method to produce energy^41^. Interestingly, in non-cancerous cells the addition of transforming growth factor beta (TGF-β) will cause the down-regulation of *c-myc* and stop cell proliferation^36^, but once the cancerous phenotype is already established, TGF-β is unable to stop them from proliferating. In summary, this data supports the idea that suspension Vero cells are becoming quiescent or senescent cells by using fatty acid beta oxidation, along with the large doubling times and down-regulated cell cycle genes. A possible method to overcome this senescence may be to up-regulate key genes such as *c-myc*^36^, *bcl2*^43^, or the addition of TGF-β^44^.

### Upregulation of kidney related genes

**Figure 6:**
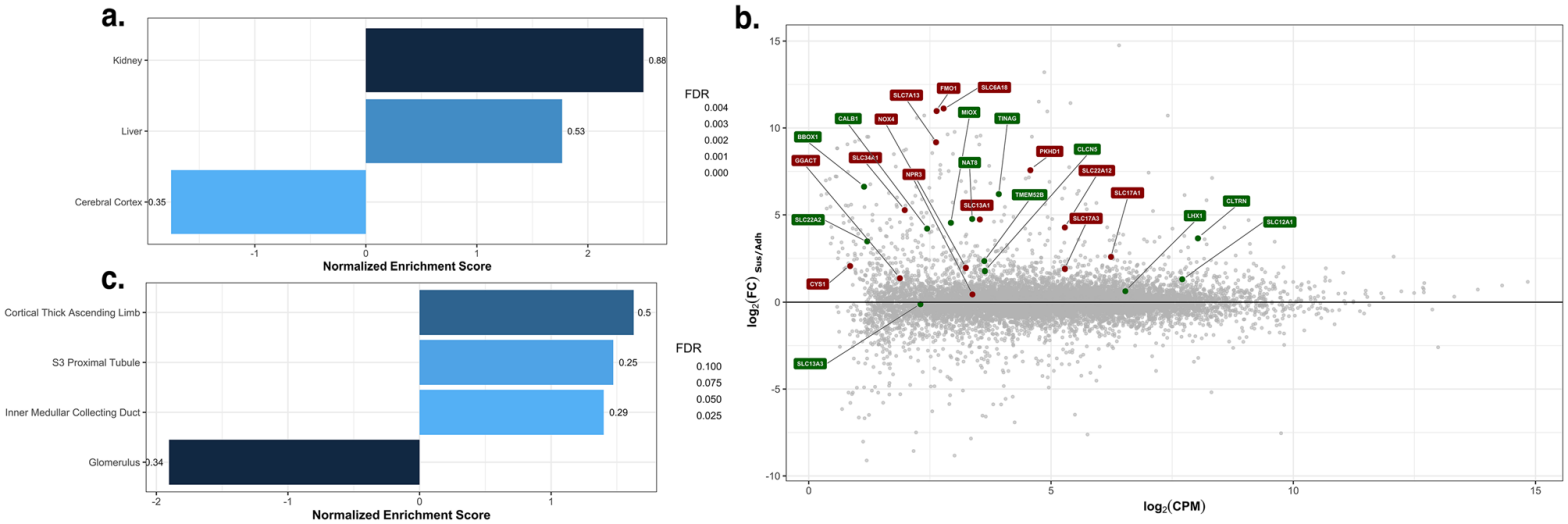
(**a**) HPA tissue related gene set enrichment analysis of Sus_CDM2 samples compared to Adh_CDM2 samples (FDR < 0.05). Tissue profiles were developed with the package TissueEnrich. Only tissue enriched genes were selected for the profiles, and only profiles with size of at least 5 specific genes were considered for the analysis. The proportion of the genes related to that profile and contributing to the enrichment scores are annotated at the side of the bars. The coloring of the bars depicts the FDR values according to the legend on the right side of the figure. (**b**) MD plot of identified kidney enriched genes with LFC and log(CPM) values. Green and red labels renal tubules or kidney associated genes according to Human Protein Atlas database, respectively. (**c**) Identification of renal tubule segment specific expression pattern. Segment related gene set enrichment analysis of Sus_CDM2 samples compared to Adh_CDM2 samples (FDR < 0.1).

An enrichment map for up-regulated GO-terms for biological processes showed three large clusters of upregulated gene sets (Fig. 4). These gene sets are related to ion and small organic molecule transport as well as amino acids and lipid metabolism. To identify if the changes in the Sus group were connected to tissue specific pattern and tissue profiles for enriched genes were defined from the human protein atlas (HPA, available from http://www.proteinatlas.org) RNA-seq data^45^. The significant profiles (p-adj < 0.05) of the gene set enrichment analysis with the tissue profiles can be seen in Fig. 6a, which shows that kidney and liver enriched genes were up-regulated. There were 17 genes associated with the liver expression profile and 12 of them contributed to the enrichment score, while the liver profile from HPA contains 242 enriched genes. The kidney profile contains 59 genes from which 25 were identified in the dataset and 22 contributed to the enrichment score. The 25 genes are further shown in the mean-difference (MD) plot in Fig. 6b. Except for *slc13a3*, *npr3*, and *lhx1*, all genes were significantly up-regulated with an FDR < 0.001. Since Vero cells were originally derived from a kidney, and the expression profile appears to specifically match the HPA kidney profile, further analysis was done on kidney-related genes.

Based on this up-regulation of renal tubule associated genes, expression changes were investigated to see if they may be related to even more specific segments of the renal tubule. To build profiles for the different segments bulk RNA-seq of dissected rat kidneys were used^46^. Thirteen segment profiles were built with TissueEnrich of which eight passed the threshold of three detected genes in the RNA-seq dataset. The results are summarized in Fig. 6c (FDR threshold of 0.1). The complete results are in Supplementary Table S9. The enrichment analysis presents an up-regulation of multiple sets referring to different parts of the renal tubule with proximal tubule segment S3, cortical thick ascending limb which is part of the Loop of Henle as well as the inner medullar part of the collecting duct (IMCD). In contrast, only the glomerulus related gene set was detected as downregulated. From the 25 genes that were identified from the dataset, twelve of these genes are specific for renal tubule according to HPA (red points in Fig. 6b). Together, these findings suggest a cellular change towards renal tubule like expression patterns during the adaption to suspension conditions used in this study.

Additionally, the analysis of transcription factor target gene sets revealed the up-regulation of multiple kidney associated transcription factors (Supplementary Table S10). The three strongest enriched genes sets were *foxi1*, *pax2*, and *hnf4a*. The *foxi1* gene is known to be important in regulating the expression of vacuolar-type H^+^-ATPase subunits in the kidney collecting duct^47^; while, *pax2* is an important factor for nephron differentiation in kidney development^48^. The main function of *hnf4a* is the control over the expression of drug metabolizing enzymes and transporters in the proximal tubule^49^. The regulatory network of these transcription factors strongly supports the directed changes towards renal tubule cells.

### Vero transition to renal tubule-like cells

#### Membrane transporter

**Figure 7:**
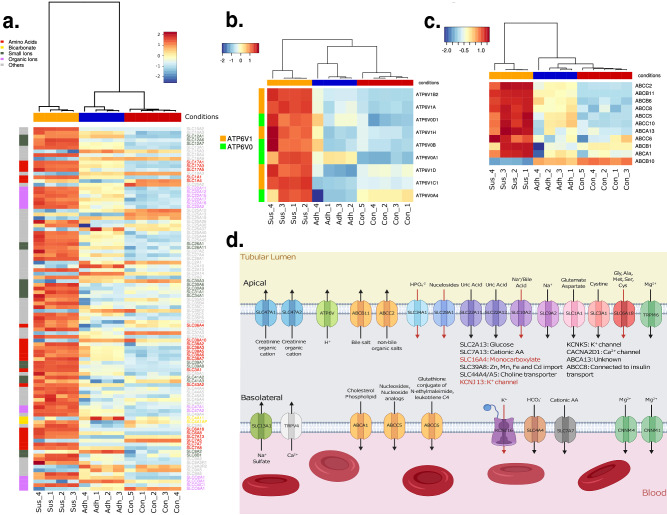
(**a**) heatmap of differentially regulated solute carrier. (**b**) heatmap of differentially regulated subunits of V-type proton ATP (ATP6V). (**c**) heatmap of differentially regulated ABC-transporter. (**d**) Scheme of the significantly up regulated membrane transporter with the target molecules and the direction of transport in a nephron. The transporters whose location are unknown are mentioned in the center of the image. Red colored arrows or gene names indicate an LFC > 5 (Created with Biorender.com).

A closer investigation of common renal tubule functions reveals even more similarities with the Sus samples. Membrane transport proteins like those from the solute carrier (SLC) superfamily are important in renal tubule cells to transport diverse molecules, from ions to lipids or amino acids, to and from the bloodstream or tubular lumen. 246 genes of this superfamily were identified as expressed. The heatmap in Fig. 7a shows the changes for solute carriers. The increased expression of many SLC genes indicate a change of the Sus cells to increased absorption and secretion like renal tubule cells.

V-ATPases form another group of transporters whose expression increased. V-ATPase are proton pumps, which contain two domains. The heatmap in Fig. 7b emphasizes the strong up-regulation of subunits for these domains in Sus samples, especially for *atpv6v1*, which is an ATP hydrolysis domain. For *atp6v1*, five of seven subunits are upregulated whereas for *atp6v0*, two of the eight are up-regulated. The clustering of the samples shows again a strong difference between Sus samples against the other samples. V-ATPase is important for proton secretion into the tubular lumen and thus urine acidification, and it is located in the apical membrane^50,51^.

A similar pattern can be seen for drug secreting ATP-binding cassette (ABC) transporters. Ten members, including *abca*, *abcb*, and *abcc* were upregulated. Again, the transporters are strongly upregulated in Sus samples as can be seen in Fig. 7c. The heatmap also shows that the increase in expression occurred progressively from Con to Adh to Sus cells. In renal tubules, ABC transporters help to secrete drugs as well as a range of macromolecules from lipids to bile salts or insulin into the tubular lumen^52–54^.

To study the transport direction, the renal tubule associated carriers are represented in Fig. 7d with substrate and orientation. The diagram shows a strong direction for transport from many molecules into the cell and from there into the blood stream. The transported target molecules that are facilitated from the highly expressed genes that are also associated with transport from the lumen are diverse and include bicarbonate, uric acid, magnesium ions and amino acids. Although the Sus samples had no separation between a potential blood stream and a tubular lumen, the RNA-seq data show an up-regulation of direction-specific transmembrane transporters for Mg^2+^ or amino acids (Fig. 7d). This suggests that when these Vero cells were placed in suspension, the physiological conditions caused the cells to revert to a more kidney-like cell that focused on filtering the medium rather than proliferating.

#### Barrier function between tubular lumen and blood stream

**Figure 8:**
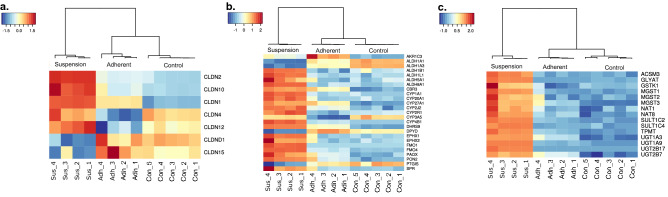
Heatmap of differentially regulated genes. The x-axis shows the samples and the y-axis represents the HGNC symbols. The coloring of the heatmap represents the standardized, normalized expression log2CPM values according to the legend at the top left side of each heatmap. (**a**) Heat map of tight junctions associated claudins. (**b**) Heatmap of differentially regulated drug metabolism genes for phase 1 modification. (**c**) Heatmap of differentially regulated drug metabolism genes for phase 2 conjugation.

To promote single cell suspension, CDM2 had low amounts of calcium and magnesium to prevent calcium-dependent cell adhesion molecules from properly functioning. Many cell adhesion molecules require calcium or magnesium to be functional such as cadherins, integrins and selectins. Although, even with lower calcium and magnesium concentrations, this did not prevent all cell adhesion since there are other adhesion molecules that do not require calcium or magnesium like claudins and vascular adhesion molecules (VCAM-1). Claudins are transmembrane proteins that specifically bind cells together at tight junctions. Vero cells that were grown adherently had down-regulated expression of various claudins, whereas suspension Vero cells up-regulated many cell adhesion proteins including claudins (except *cldn15*) (Fig. 8). Tight junctions are especially important in the kidney tubule to control passive reabsorption of particular compounds between the blood stream and the tubular lumen, which also indicates that the suspension Vero cells were behaving more like senescent kidney cells^55^. The upregulation of these proteins, along with other cell adhesion molecules such as *itgb2*, *vcam1* and *jam1* may be the reason why even with low calcium and magnesium levels, the cells would still form aggregates (Fig. 2a).

#### Conclusion

A chemically defined media was designed that supports the growth of various mammalian cell lines in suspension. Vero cells were unable to effectively proliferate in suspension in this medium, and transcriptomic work revealed that these cells were behaving more as mature kidney cells. Overall, the RNA-seq data shown here demonstrated that suspension Vero cells became senescent rather than apoptotic, and that genes associated with cell-cycle progression were down-regulated and genes associated with fatty acid beta oxidation were up-regulated. In addition to the arrest in growth, suspension Vero cells up-regulated kidney tissue-associated genes. Suspension Vero cells specifically up-regulated genes associated with solute membrane transporters and proteins that form tight junctions which is important for the epithelial barrier and function of the tubule of the kidney for filtering blood. This work offers insights as to why Vero cells have been troublesome to adapt to suspension in the past and elucidates some possible avenues to establish a robust suspension cell line to support vaccine production using Vero cells.

## Materials and methods

### Cell culture

Vero cells (CCL81 n + 51, ATCC, USA) were thawed from liquid nitrogen and maintained in DMEM/F12 (Corning, USA) + 10% FBS (Hyclone, USA) + 4 mM GlutaMax (Gibco, USA) in a humidified, 5% CO_2_ incubator at 37 °C. Cells were passaged every 2–3 days to keep confluence below 90%. CHO-K1 cells (gift from Dr. Pu Chen, University of Waterloo, Canada) were thawed from liquid nitrogen and maintained in DMEM/F12 + 10% FBS in a humidified, 5% CO_2_ incubator at 37 °C. Cells were passaged every 4–5 days to keep confluence below 90%. MDCK cells (gift from Dr. Matthew Miller, McMaster University, Canada) were thawed from liquid nitrogen and maintained in DMEM + 10% FBS in a humidified, 5% CO_2_ incubator at 37 °C. Cells were passaged every 2–3 days to keep confluence below 90%. HEK293T cells (gift from Dr. Matthew Miller, McMaster University, Canada) were thawed from liquid nitrogen and maintained in DMEM + 10% FBS in a humidified, 5% CO_2_ incubator at 37°C. Cells were passaged every 2–3 days to keep confluence below 90%.

### Media development

Unless otherwise stated, all compounds were obtained from Sigma-Aldrich. All media formulations used a DMEM/F12 base with 15 mM HEPES, without l-glutamine, l-leucine, l-lysine, l-methionine, CaCl_2,_ MgCl_2_, MgSO_4_, sodium bicarbonate, and phenol red (cat #D9785, Sigma-Aldrich, USA). The HT supplement was purchased from Gibco. To screen compounds, a Plackett–Burman style of experiments was used with 24 media samples that tested 23 different compounds. To make the media, first stock solutions of the compounds were made and then 1 L of the calcium and magnesium-free DMEM/F12 was made. 40 mL of each media sample was made by supplementing DMEM/F12 with the various compounds, without increasing the volume more than 10%. All media formulations used 0.1 mM calcium and 0.1 mM magnesium, to ensure suspension growth of the Vero cells, unless otherwise stated.

Vero cells were slowly adapted to the new media samples over 90 days in treated T25 flasks (VWR, Canada) by exchanging serum-containing media (DMEM/F12 + 10%FBS) with the new formulation in 25% increments. Once the cells were fully adapted, they were then cultured in non-treated T25 flasks (VWR, Canada) for an additional 90 days. After 180 days, the flasks were slowly shaken (40 rpm) to break up clumps. Media samples that were able to support viable cells were cultured and all others were discontinued.

### RNA Seq analysis

Vero cells were maintained in DMEM/F12 + 10% FBS, Formula 17, and Formula 17 + 1 mM calcium and 1 mM magnesium. Cell samples were collected for RNA extraction from approximately 3 × 10^5^ cells to 10^6^ cells. The RNA was extracted using a Total RNA Tissue Kit (Roche) and samples were stored at – 80 °C. The cDNA libraries were prepared and sequenced at the Centre for Applied Genomics, Sick Kids Hospital, Toronto, Canada. The sequenced reads were received in FASTQ format with > 15 Million paired end reads of 125-bp length per sample. Reads were aligned with the STAR aligner v2.6.0^56^ to the Vervet-AGM genome assembly 43 (ChlSab1.1: GCA_000409795.2) from Ensembl release 96.1. The gene expression level was quantified with RSEM v1.3.1^57^ using default settings. The quantified reads for each gene per sample were then further analyzed in RStudio^58^ with the package edgeR^59^. For pathway analysis, the Vervet-AGM gene annotations from Ensembl were changed to human homologs with HGNC symbols annotation using biomaRt^60^.

### Gene set enrichment analysis

Gene set enrichment was performed using the gene set enrichment analysis (GSEA) application^61^. The gene list from edgeR was ordered by the − log10(p-values) multiplied with the sign of the LFC. The first tested gene sets were from biological processes from Gene Ontology (GO)^62^. The enrichment results for GO sets were visualized in Cytoscape^63^ with the package Enrichment map^61^. For the enrichment map, a false discovery rate (FDR) threshold of 5% was applied to filter the enriched gene set list, and the overlap of the gene sets was calculated with a combination of Jaccard and Overlap similarity coefficient of 20 to 80 and a threshold of 0.6. To obtain tissue type specific gene sets, RNA-seq data from Human Protein Atlas (HPA)^45^ for 35 tissues were analyzed with TissueEnrich. For the GSEA of both sets, the minimum identified numbers of genes were set to five. Transcription factor target gene sets from GTEx^64^ were tested for enrichment to identify regulatory patterns in the data sets. The gene sets contained 1607 transcription factors, each with 300 target genes. The GSEA was performed using default settings.

### Housekeeping gene expression for quality control

A Pearson correlation of housekeeping genes was performed for all samples against all samples. Housekeeping genes were defined based on the investigation by Eisenberg and Levanon^65^ with 3122 housekeeping genes identified as expressed in our RNA-seq data. The correlation was calculated with the R package corrplot^66^. A positive expression correlation of r > 0.90 between samples is indicative of biologically intact samples. The high correlation value shows that the cells in each treatment had similar expression levels of housekeeping genes.

### RNA-seq heatmaps

Heatmaps were generated with the package heatmap3^67^. The weighted trimmed mean of M values (TMM) standardized, normalized log2 counts per million (CPM) gene list were filtered with an FDR threshold of 0.1%. The Pearson distance matrix was calculated and clustered by an agglomerative hierarchical clustering algorithm with the complete-linkage method.

## Supplementary Information

Supplementary Information 1.

Supplementary Table S3.

Supplementary Information 3.

Supplementary Table S8.

Supplementary Table S9.

Supplementary Table S10.
