# Supplementary material for: Vero cells gain renal tubule markers in low-calcium and magnesium chemically defined media
Source: Sci Rep. 2022 Apr 13;12:6180. doi: 10.1038/s41598-022-10221-z (PMC9008052; doi:10.1038/s41598-022-10221-z)
Supplement: Supplementary file 1 — Supplementary Information 1. [file 41598_2022_10221_MOESM1_ESM.docx]

**Supplementary Information**

**Vero cells gain renal tubule markers in low-calcium and magnesium chemically defined media**

Megan Logan^+,1^, Karsten Rinas^+,2^, Brendan McConkey^2^, Marc G. Aucoin^1,*^

^1^Department of Chemical Engineering, University of Waterloo

^2^Department of Biology, University of Waterloo

^+^Should be considered joint first authors

***corresponding author:** Marc G. Aucoin

Department of Chemical Engineering

University of Waterloo

Waterloo, ON

CANADA N2L 3G1

Tel: 1-519-888-4567 x36084

Fax:1-519-888-4347

E-mail address: [maucoin@uwaterloo.ca](mailto:maucoin@uwaterloo.ca)

**Supplementary Table S1**. List of compounds that were tested in the Plackett-Burman experiments to formulate a suspension media for Vero cells. A review of journal articles and patents for serum-free mammalian media was conducted to create a design space for media development.

| Amino Acids | Metals | Vitamins | Lipids, Fatty acids and Steroids | Other |
| --- | --- | --- | --- | --- |
| Arginine  Asparagine  Aspartate  Cysteine  Glutamine  Isoleucine  Leucine  Lysine  Methionine  Ornithine  Serine  Threonine  Tryptophan | CaCl_2_  CoCl_2_  CuCl_2_  Fe·Citrate  MnSO_4_  MgCl_2_  MgSO_4_  NaSiO_3_  Na_2_SeO_3_  NiSO_4_  (NH_4_)_6_Mo_7_O_24_  SnCl_2_  V_2_O_5_ | Ascorbic acid  α-tocopherol  Choline Chloride  D-Pantothenate ½ Calcium  D-Biotin  Folic Acid  Myo-inositol  Nicotinic Acid  Niacinamide  Pyridoxine  Thiamine  Vitamin B_12_ | Cholesterol  Arachondic Acid  Myristic Acid  Linoleic Acid  Linolenic Acid  Oleic Acid  Palmitic Acid  Stearic Acid  Tween-80  Hydrocortisone  Dexamethasone | Glucose  Bicarbonate  rEGF  IGF-1  Dextran Sulfate  Poloxamer 188  Ethanolamine  Adenine  Guanosine  Hypoxanthine  Thymidine  Uridine  Glutathione  Putrescine |

**Supplementary Table S2** Summary of RNA-seq data collected of Vero cell lines in different conditions. *Con: control cells in FBS containing media. ^#^ Adh: adherent Vero cells in defined media. ^$^Sus: suspension Vero cells in defined media

| Sample: | Con1* | Con2 | Con3 | Con-4 | Con-5 | Adh-1^#^ | Adh-2 | Adh-3 | Adh-4 | Sus-1^$^ | Sus-2 | Sus-3 | Sus-4 |
| --- | --- | --- | --- | --- | --- | --- | --- | --- | --- | --- | --- | --- | --- |
| Input reads  (in mil) | 20.6 | 20.5 | 21.6 | 20.4 | 20.7 | 22.1 | 15.0 | 22.0 | 23.5 | 21.4 | 20.9 | 18.6 | 22.8 |
| Uniquely Aligned   (in mil) | 19.9 | 19.8 | 20.8 | 19.7 | 19.8 | 21.3 | 14.5 | 21.1 | 22.4 | 20.6 | 20.1 | 17.9 | 21.9 |
| Uniquely mapped reads % | 96.17% | 96.24% | 96.15% | 96.17% | 96.04% | 96.17% | 96.49% | 96.44% | 95.40% | 96.03% | 96.18% | 96.20% | 96.16% |
| Number of reads mapped to multiple loci | 692,601 | 682,474 | 732,797 | 686,586 | 711,826 | 731,493 | 444,772 | 658,007 | 909,751 | 732,510 | 684,189 | 603,512 | 758,962 |
| % of reads mapped to multiple loci | 3.35% | 3.33% | 3.38% | 3.35% | 3.45% | 3.31% | 2.96% | 3.00% | 3.87% | 3.42% | 3.27% | 3.25% | 3.33% |
| Detected genes | 14,656 | 14,670 | 14,723 | 14,610 | 14,462 | 14,809 | 14,433 | 14,782 | 14,775 | 14,650 | 14,633 | 14,539 | 14,711 |

S3 Excel Sheet CPM values after filtering

S4-S7 Excel Sheet DEG analysis results

S8 Excel Sheet GSEA of GO BP gene sets

S9 Excel Sheet GSEA of renal tubule specific gene sets

S10 Excel Sheet GSEA of transcription factor specific gene sets

**Supplementary Figures**


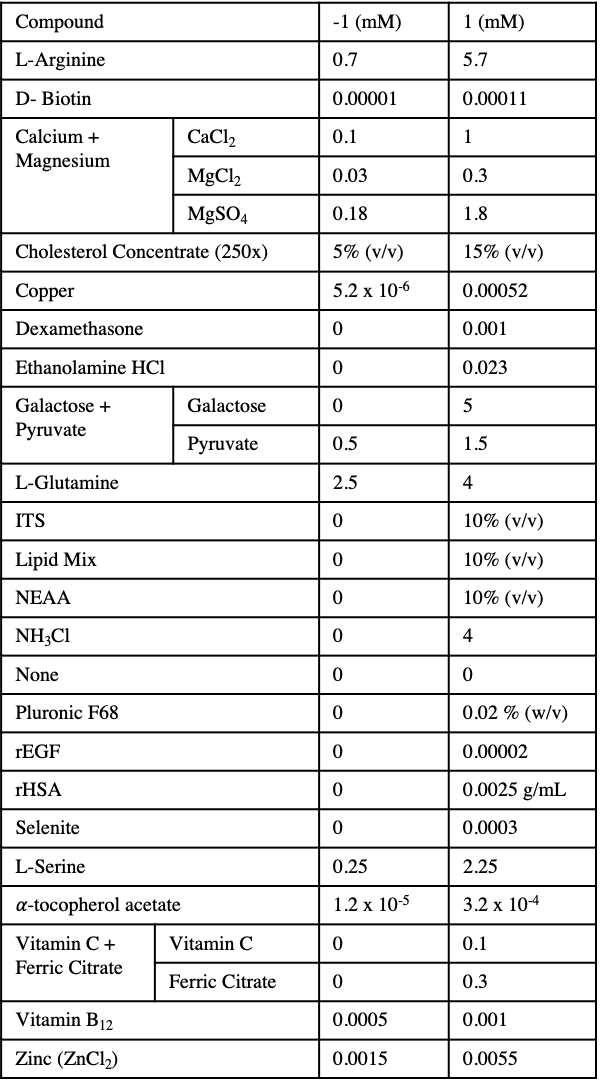
**Supplementary Figure S1**. Plackett Burman screening experiment #1. The box plots that show the effect of each media component on viable cell density (VCD) for a Plackett-Burman experiment with 23 factors in 24 runs. This experiment used a basal media with low calcium and magnesium to encourage suspension growth and spiked in components at various concentrations that are listed in the table to the right.


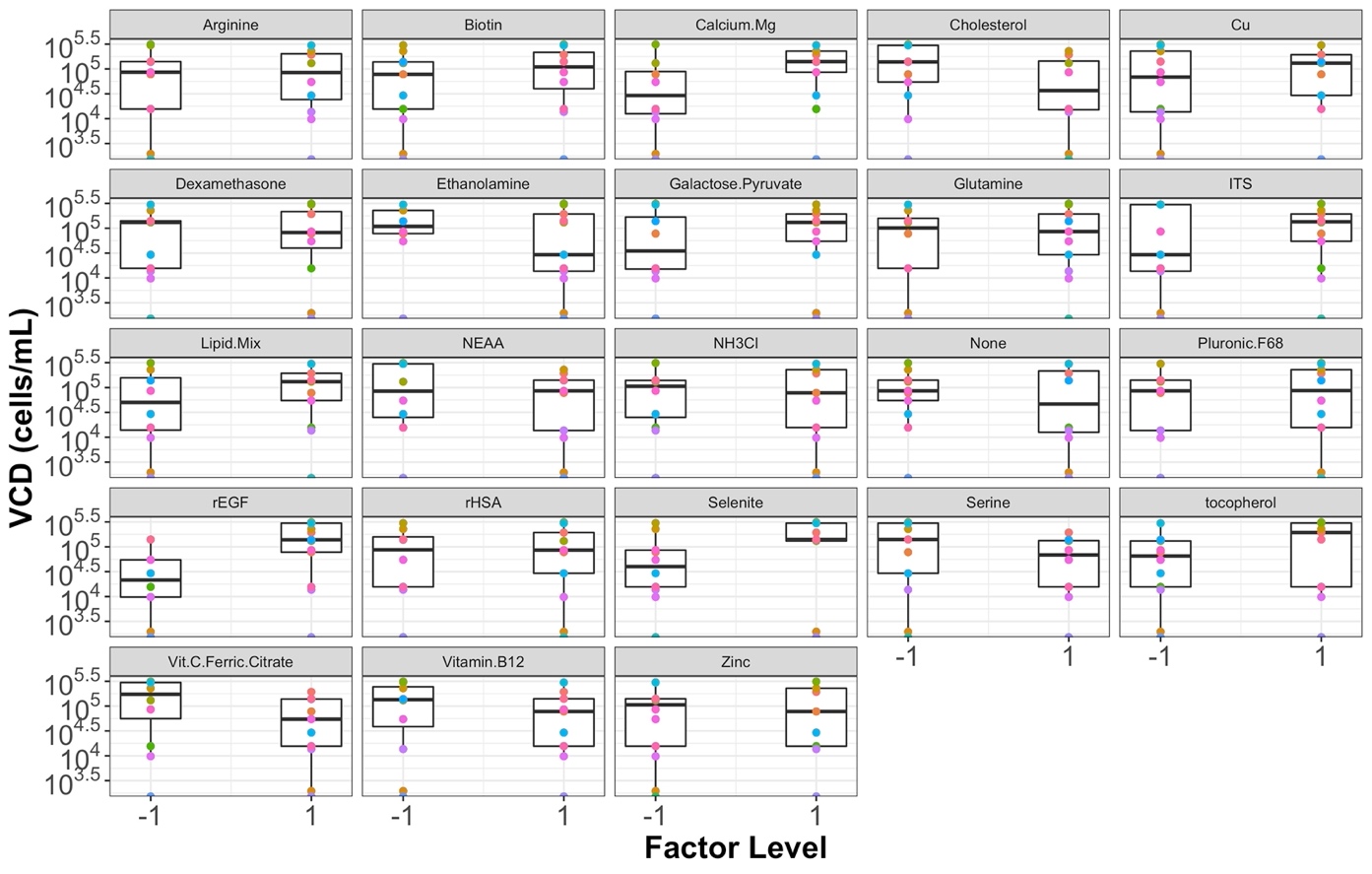


Plackett-Burman experiment #1 results (cont.)

| term | Coeffic | t value | p.value |
| --- | --- | --- | --- |
| (Intercept) | 85412.5184 | 13.2616929 | 1.15E-17 |
| xrEGF | 64408.7617 | 10.0005156 | 2.53E-13 |
| Calcium.Mg | 33956.1451 | 5.27224789 | 3.17E-06 |
| xSerine | -31658.382 | -4.9154825 | 1.07E-05 |
| xVit.C.Ferric.Citrate | -29888.917 | -4.640744 | 2.71E-05 |
| xCholesterol | -28226.806 | -4.3826742 | 6.36E-05 |
| xITS | 23195.3042 | 3.60145102 | 0.00074863 |
| xPluronic.F68 | 22067.8211 | 3.4263908 | 0.00126307 |
| xLipid.Mix | 16871.0418 | 2.61950566 | 0.01175218 |
| Selenite | 16033.6804 | 2.48949159 | 0.01631041 |
| xtocopherol | 14727.9863 | 2.28676118 | 0.02666185 |
| xNH3Cl | 13128.6021 | 2.03843058 | 0.04703625 |
| xVitamin.B12 | -13106.015 | -2.0349235 | 0.04740159 |
| xZinc | -12785.832 | -1.9852099 | 0.052851 |
| xGalactose.Pyruvate | 11826.3978 | 1.83624203 | 0.0725194 |
| xEthanolamine | 11648.8161 | 1.80866956 | 0.07676845 |
| Cu | -11463.922 | -1.7799616 | 0.08141298 |
| xNEAA | -11166.734 | -1.7338184 | 0.0893698 |
| xrHSA | 9698.12177 | 1.5057923 | 0.13867344 |
| xGlutamine | 9515.08988 | 1.47737361 | 0.14610723 |
| xBiotin | 7559.76845 | 1.17377792 | 0.24627526 |
| xArginine | -5414.4765 | -0.8406862 | 0.40469221 |
| xNone | -4305.5378 | -0.6685053 | 0.50701338 |
| xDexamethasone | 1533.06367 | 0.23803325 | 0.81286851 |

**Supplementary Figure S2**. Plackett Burman screening experiment #2. The box plots that show the effect of each media component on viable cell density (VCD) for a Plackett-Burman experiment with 23 factors in 24 runs. This experiment used a basal media with low calcium and magnesium to encourage suspension growth and spiked in components at various concentrations that are listed in the table to the right.


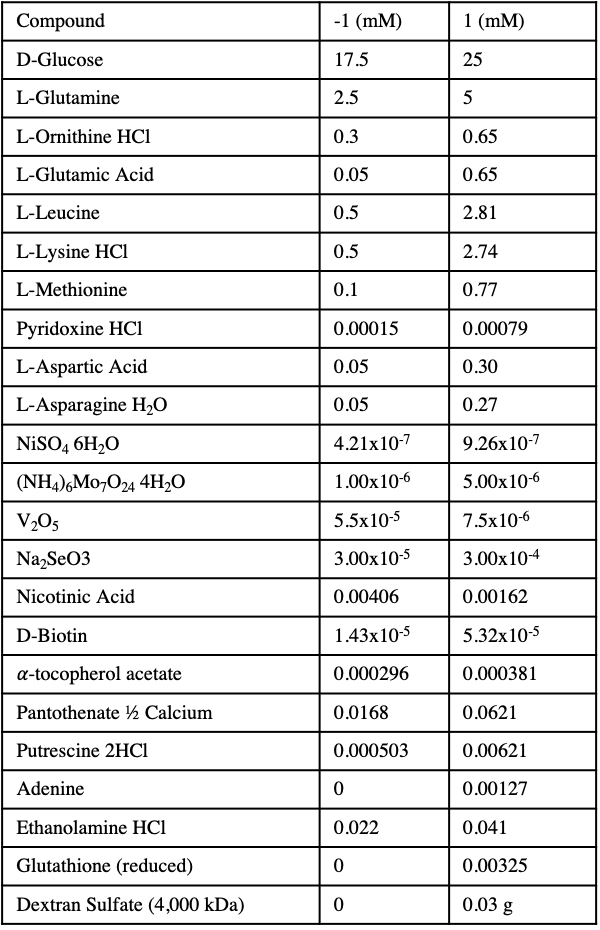

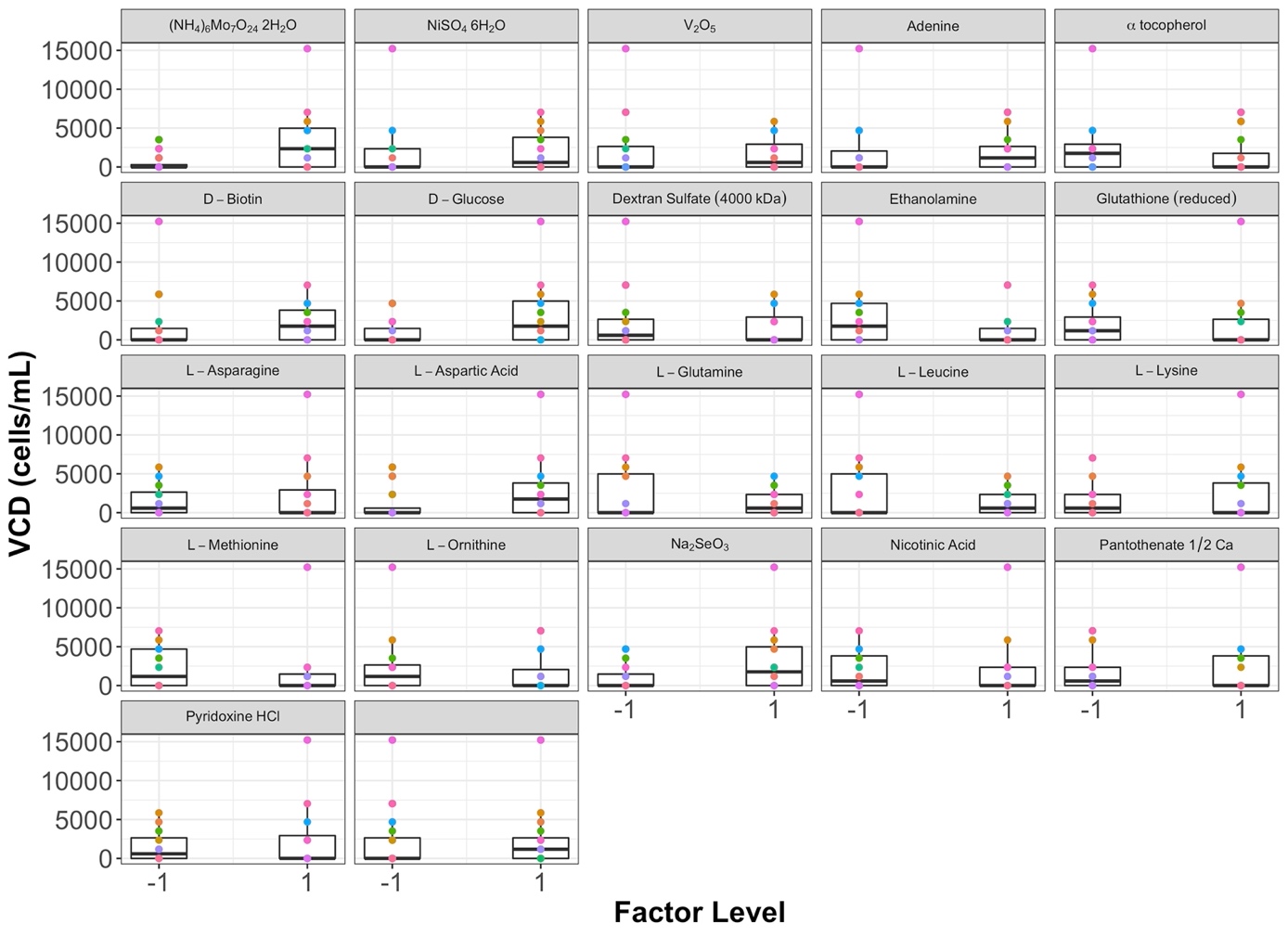


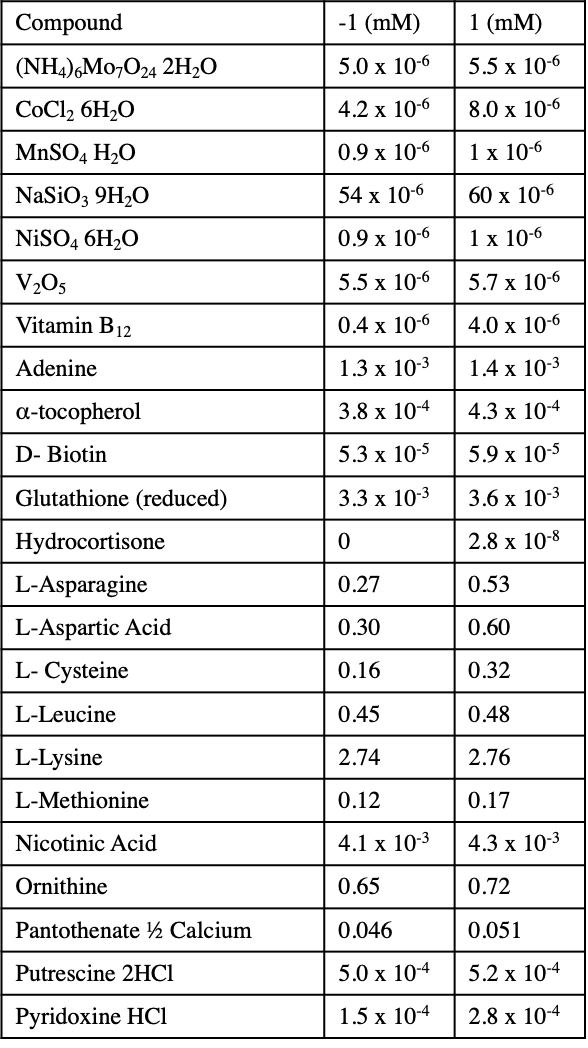
**Supplementary Figure S3**. Results from Plackett-Burman experiment #3. The box plots that show the effect of each media component on growth rate for a Plackett-Burman experiment with 23 factors in 24 runs. This experiment used a basal media with low calcium and magnesium to encourage suspension growth and spiked in components at various concentrations that are listed in the table to the right.


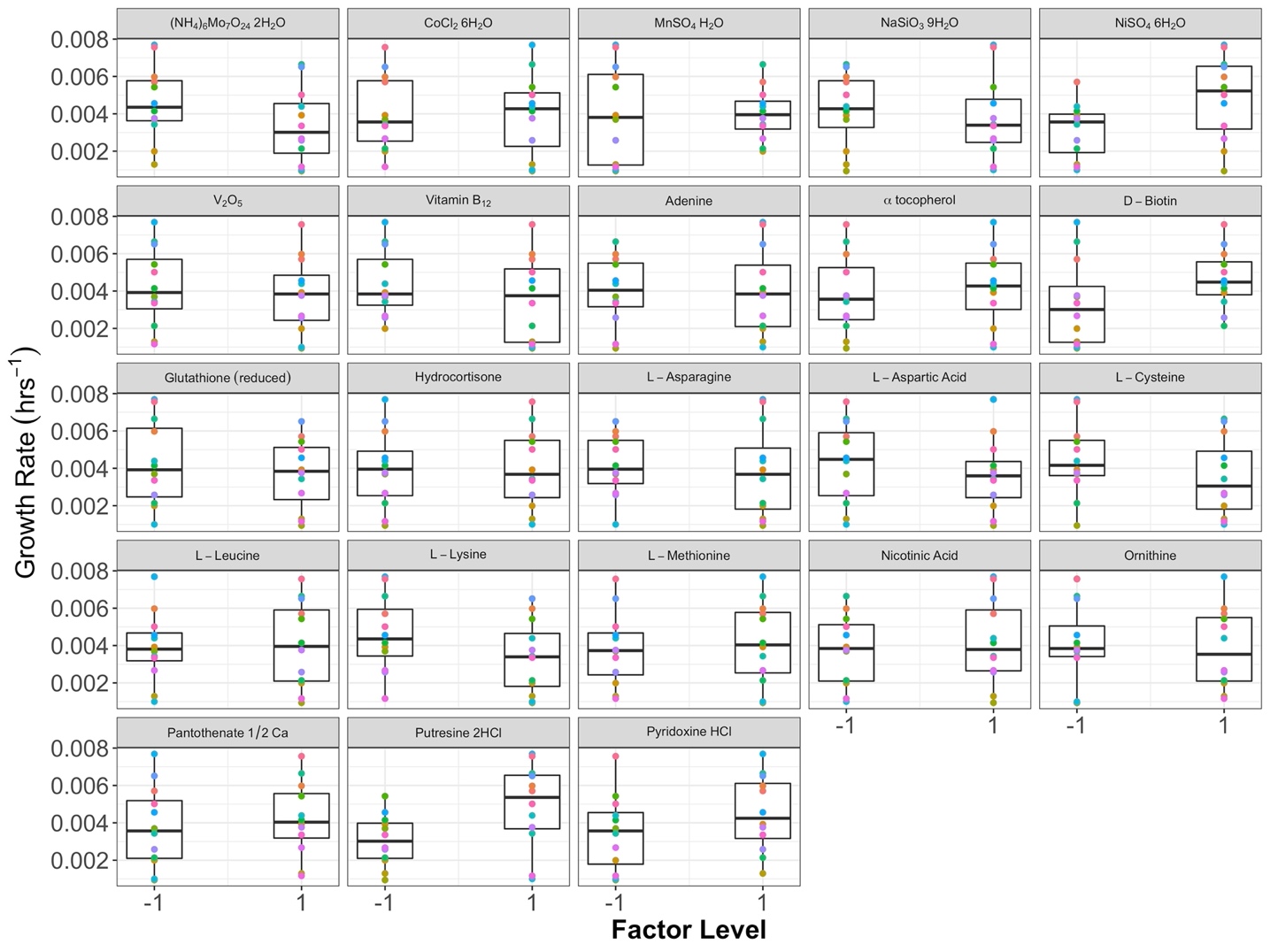


Plackett-Burman experiment #3 results (cont.)

| term | estimate | std.error | statistic | p.value |
| --- | --- | --- | --- | --- |
| (Intercept) | 0.0053149 | 0.00010526 | 50.4929462 | 0 |
| nicotinic.acid | 0.00060403 | 0.00010526 | 5.73845378 | 1.03E-08 |
| Glutathione | -0.0005816 | 0.00010526 | -5.5253558 | 3.50E-08 |
| CoCl2.6H2O | 0.00055397 | 0.00010526 | 5.26281054 | 1.49E-07 |
| V2O5 | -0.0004519 | 0.00010526 | -4.2929282 | 1.81E-05 |
| Asparagine | -0.0004258 | 0.00010526 | -4.0454226 | 5.32E-05 |
| Pantothenate.hemicalcium | -0.0004227 | 0.00010526 | -4.0153947 | 6.04E-05 |
| Biotin | 0.00032498 | 0.00010526 | 3.08741161 | 0.00203307 |
| NaSiO3.9H2O | -0.0003201 | 0.00010526 | -3.0412689 | 0.00237128 |
| Methionine | -0.0003093 | 0.00010526 | -2.938073 | 0.00332168 |
| Aspartic.Acid | -0.000252 | 0.00010526 | -2.3942782 | 0.01669942 |
| Lysine | 0.00024225 | 0.00010526 | 2.30144346 | 0.02141833 |
| vitamin.b12 | 0.00022907 | 0.00010526 | 2.1762106 | 0.02959852 |
| adenine | -0.000227 | 0.00010526 | -2.1567341 | 0.03108648 |
| Putresine.2.HCl | 0.00021198 | 0.00010526 | 2.01382169 | 0.04409587 |
| Leucine | 0.00020717 | 0.00010526 | 1.96818117 | 0.04911696 |
| X.NH4.6Mo7O24.4H2O | -0.0002047 | 0.00010526 | -1.9448526 | 0.05186353 |
| pyridoxine | -0.0001388 | 0.00010526 | -1.3181634 | 0.18752531 |
| Ornithine | 0.00013809 | 0.00010526 | 1.31184313 | 0.18964923 |
| MnSO4.H20 | 9.12E-05 | 0.00010526 | 0.86611538 | 0.38647944 |
| Hydrocortisone | 7.59E-05 | 0.00010526 | 0.72147892 | 0.47065757 |
| NiSO4.6H2O | -7.41E-05 | 0.00010526 | -0.7042809 | 0.48129933 |
| Cysteine | 7.39E-05 | 0.00010526 | 0.70207048 | 0.48267654 |
| a.tocopherol | -5.89E-05 | 0.00010526 | -0.5591803 | 0.57607034 |


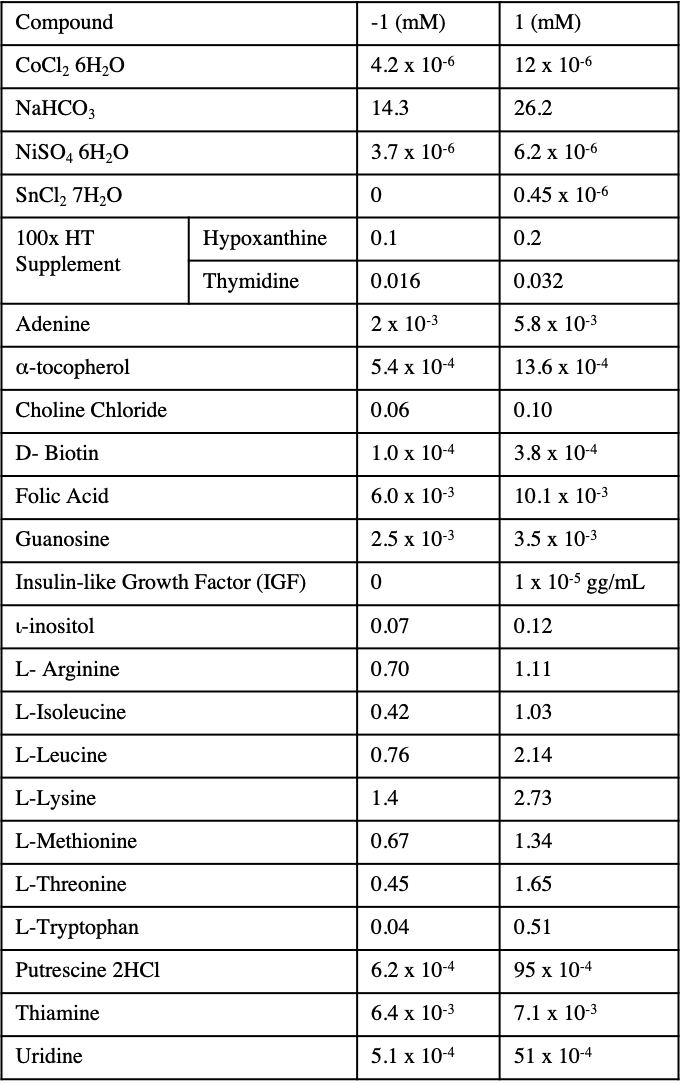

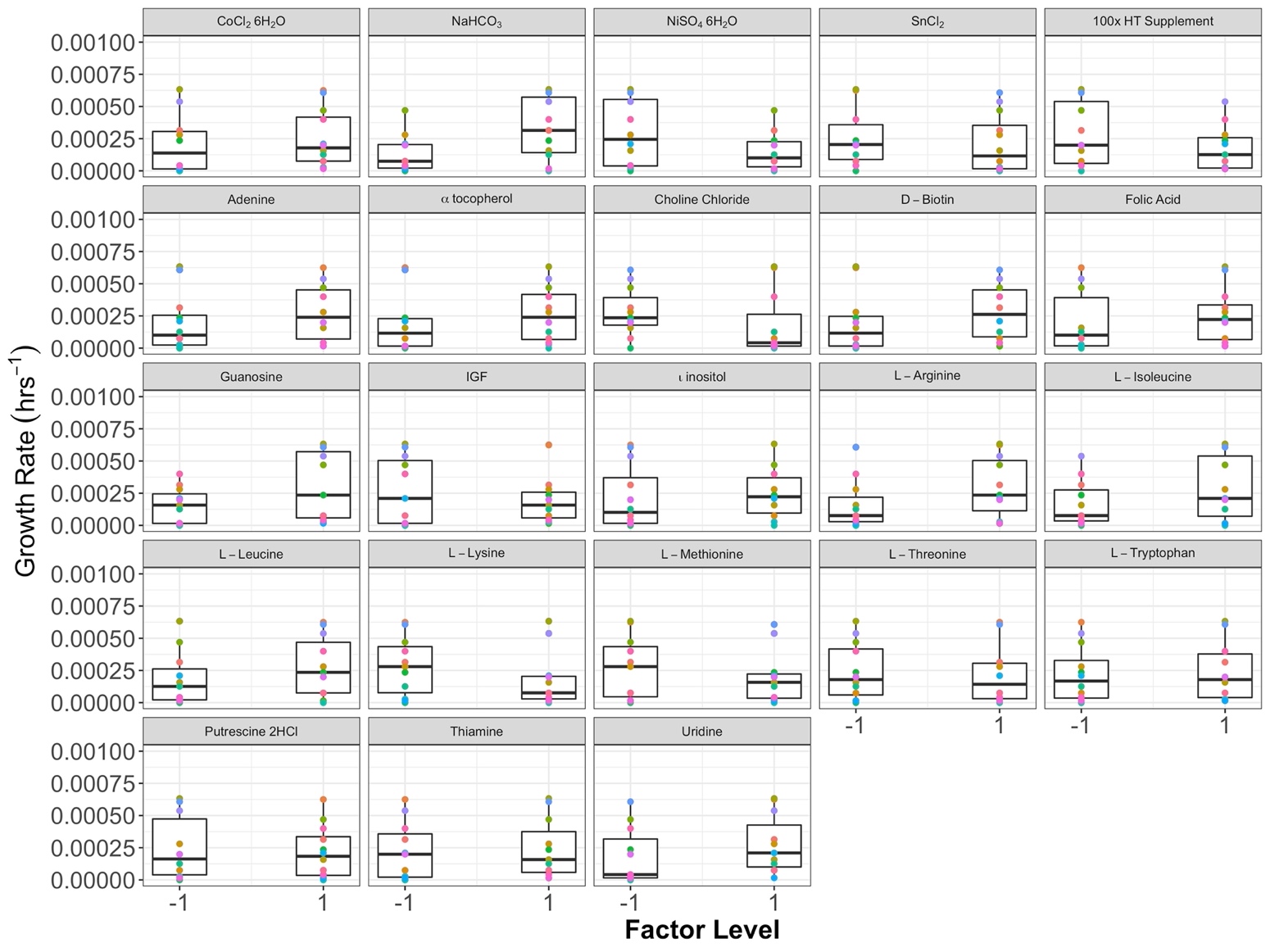
**Supplementary Figure S4**. Results from Plackett-Burman experiment #4. The box plots that show the effect of each media component on growth rate for a Plackett-Burman experiment with 23 factors in 24 runs. This experiment used a basal media with low calcium and magnesium to encourage suspension growth and spiked in components at various concentrations that are listed in the table to the right.

Results from Plackett-Burman experiment #4 (cont.)

| term | estimate | std.error | statistic | p.value |
| --- | --- | --- | --- | --- |
| (Intercept) | 0.00517709 | 0.00039505 | 13.1050307 | 8.01E-28 |
| Folic.Acid | 0.00182655 | 0.00039505 | 4.62362997 | 7.28E-06 |
| CoCl2 | -0.0017617 | 0.00039505 | -4.4595707 | 1.46E-05 |
| Putrescine | -0.001456 | 0.00039505 | -3.6857319 | 0.00030345 |
| Thiamine | -0.0012284 | 0.00039505 | -3.109539 | 0.00218603 |
| alpha.tocopherol | 0.00119018 | 0.00039505 | 3.01275403 | 0.00297069 |
| NISO4 | -0.0011176 | 0.00039505 | -2.8291026 | 0.00521006 |
| NaHCO3 | 0.00100687 | 0.00039505 | 2.54873566 | 0.01166558 |
| Choline.Chloride | 0.00095685 | 0.00039505 | 2.42211114 | 0.01644517 |
| Lysine | 0.00080237 | 0.00039505 | 2.03107758 | 0.04375149 |
| HT.Supplement..100x..gibco | 0.00078341 | 0.00039505 | 1.98307979 | 0.04891405 |
| myo.inositol | -0.0005752 | 0.00039505 | -1.4560188 | 0.14716895 |
| Uridine | -0.0005572 | 0.00039505 | -1.4103515 | 0.16020083 |
| Leucine | -0.0003896 | 0.00039505 | -0.9863024 | 0.32533872 |
| Arginine | -0.0003764 | 0.00039505 | -0.952907 | 0.34194406 |
| IGF | -0.0002969 | 0.00039505 | -0.7515782 | 0.45330858 |
| Guanosine | 0.00028064 | 0.00039505 | 0.71040344 | 0.47839426 |
| Methionine | 0.00016824 | 0.00039505 | 0.4258746 | 0.67071957 |
| Isoleucine | 0.00016293 | 0.00039505 | 0.41243896 | 0.68051966 |
| Adenine | 0.00013229 | 0.00039505 | 0.33488322 | 0.73811196 |
| Threonine | 0.00012498 | 0.00039505 | 0.31637413 | 0.75209346 |
| D.Biotin | -9.40E-05 | 0.00039505 | -0.2378639 | 0.81226331 |
| tryptophan | 7.11E-05 | 0.00039505 | 0.17999733 | 0.85736174 |
| SnCl2 | -8.68E-06 | 0.00039505 | -0.0219814 | 0.98248766 |

**Supplementary Figure S5.** Analysis of RNA-seq data variance. **a.** two-dimensional plot of principal component analysis (PCA) for all 13 samples of the 500 most variable genes. Each symbol represents one sample. Group affiliation is shown in the legend on the right side. **b.** Pearson's correlation between all samples for 3122 detected housekeeping genes. Correlation coefficients *r*values are annotated, and colour coded according to the legend on the right side of the figure.


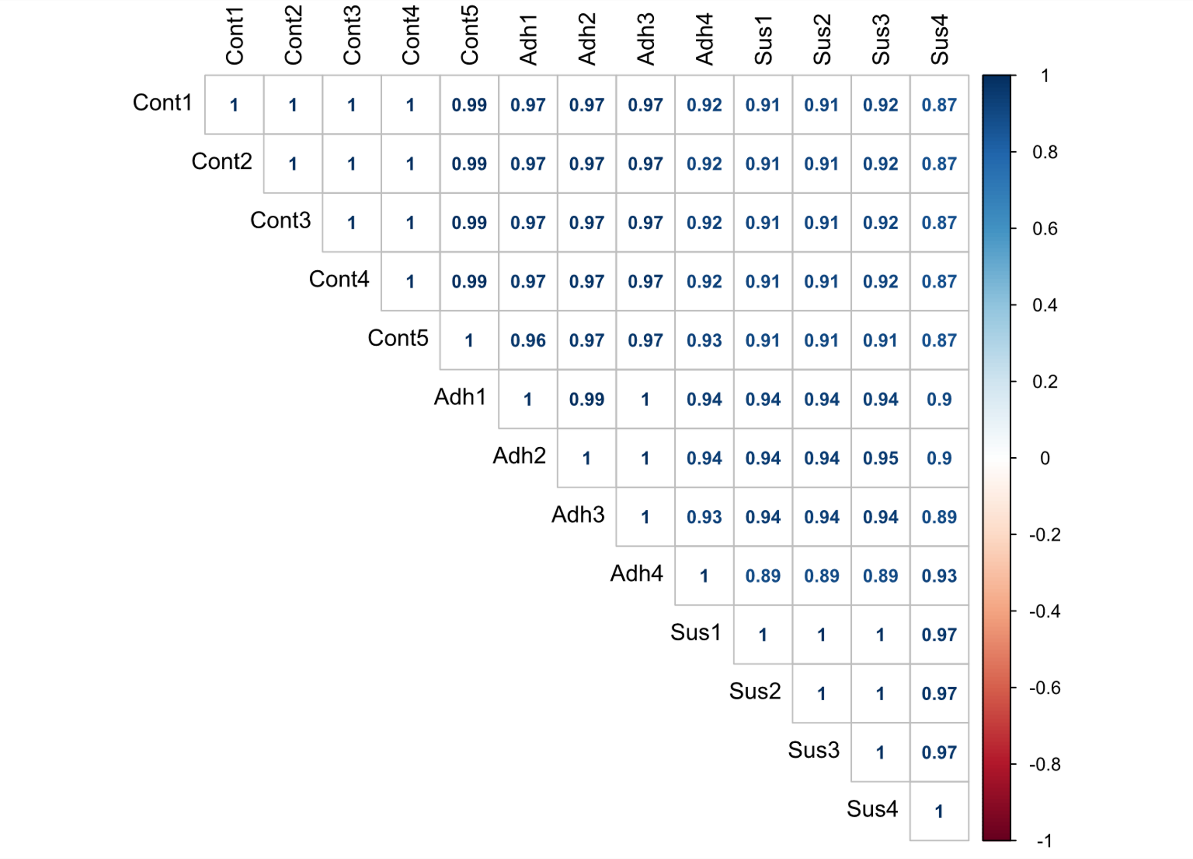

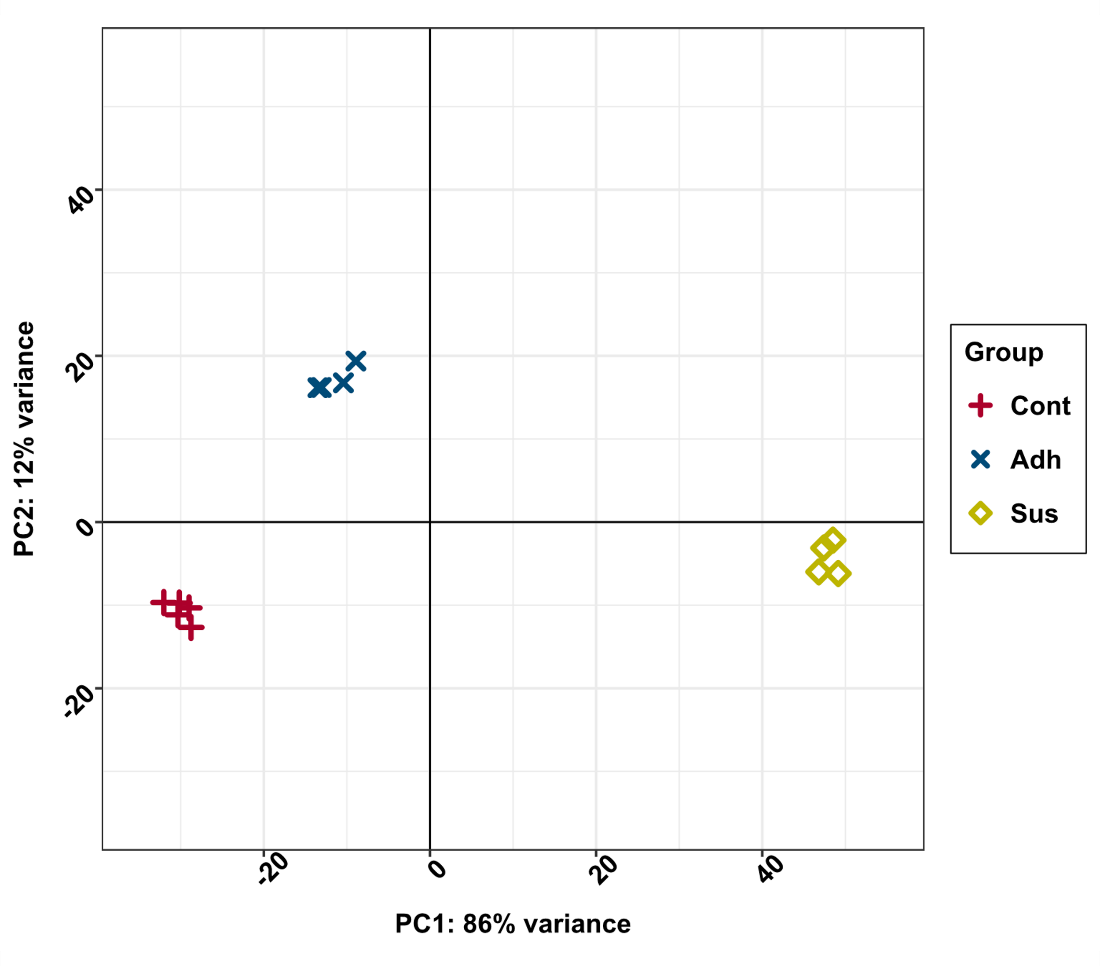


**b.**

**a.**

**b.**

**a.**

**Supplementary Figure S6.** Upregulation of fatty acid beta oxidation related genes (GO:0006635). **a.** Diagram of fatty acid beta oxidation with the main reactions and enzymes in mitochondria. Green arrows label significant upregulation of at least one gene in that reaction group with an FDR threshold of 0.001. Red arrows emphasize the fatty acid and derivatives flow through the process until Acetyl-CoA. **b.** heatmap of differentially regulated genes. The x-axis shows the samples, and the y-axis represents the HGNC symbols. Coloring of the samples is the same as in figure A. The coloring of the heatmap represents the standardized, normalized expression log2CPM values according to the legend at the top left side of each heatmap. ​

**a.**

**b.**

**
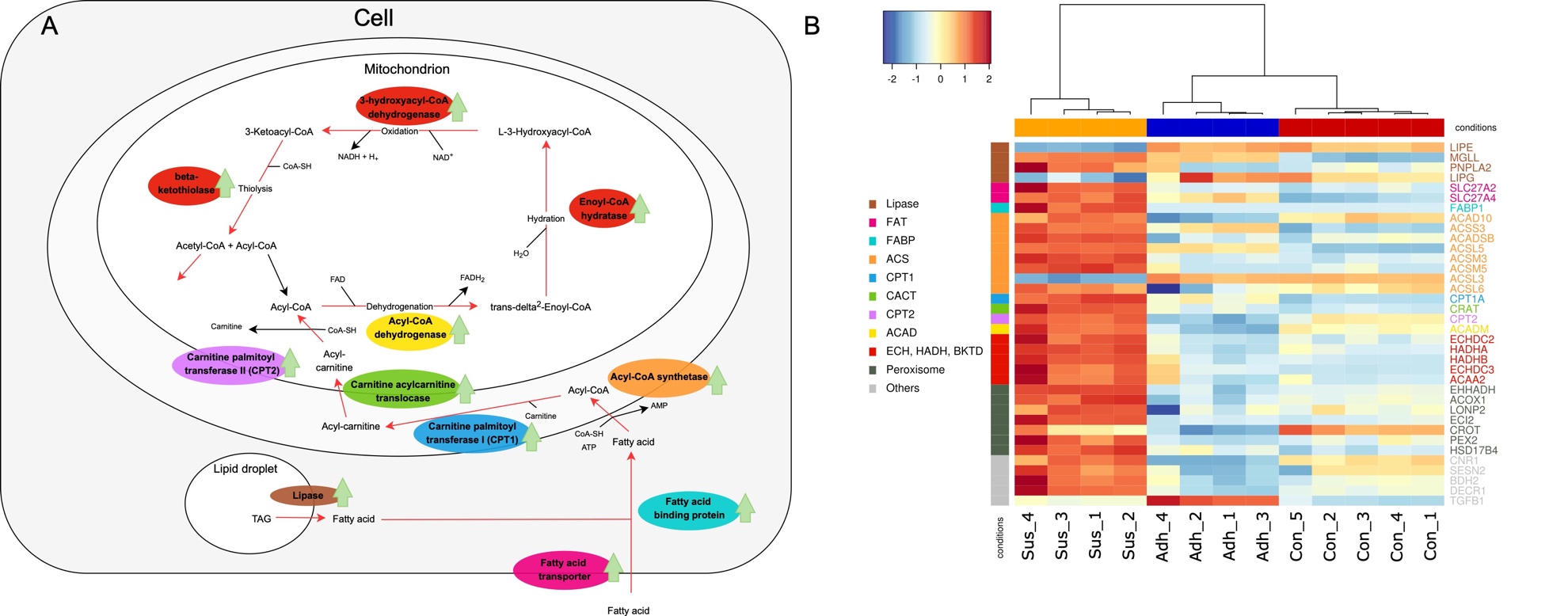
**

**Supplementary Figure S7.** Identification of directed gene expression changes caused by known transcription factors. Target gene sets were received from the GTEx dataset. The x-axis represents the regulatory transcription factor and the y-axis shows the identified target genes contributing to the leading edge of the GSEA.  The color scheme of the heatmaps depicts the LFC of the identified genes for the contrast Sus vs. Adh_CDM2. **a.** target gene heatmap of the top ten transcription factors with the lowest normalized enrichment score in the GSEA. The colors of the gene labels refer to key gene sets of the identified, down-regulated clusters from Figure 2. The Gene ontology gene set names are the following: red: Cell cycle phase transition, blue: DNA repair, purple: Chromatin organization, orange: Microtubule cytoskeleton organization, brown: mRNA metabolic process. **b.** target gene heatmap of the top ten transcription factors with the highest normalized enrichment score in the GSEA. The colors of the gene labels refer to key gene sets of the identified, up-regulated clusters from Figure 2. The Gene ontology gene set names are the following: red: Anion transmembrane transport, blue: Endoplasmic reticulum to Golgi vesicle mediated transport, purple: Cellular ion homeostasis, orange: Cellular lipid metabolic process, brown: Leukocyte mediated immunity.


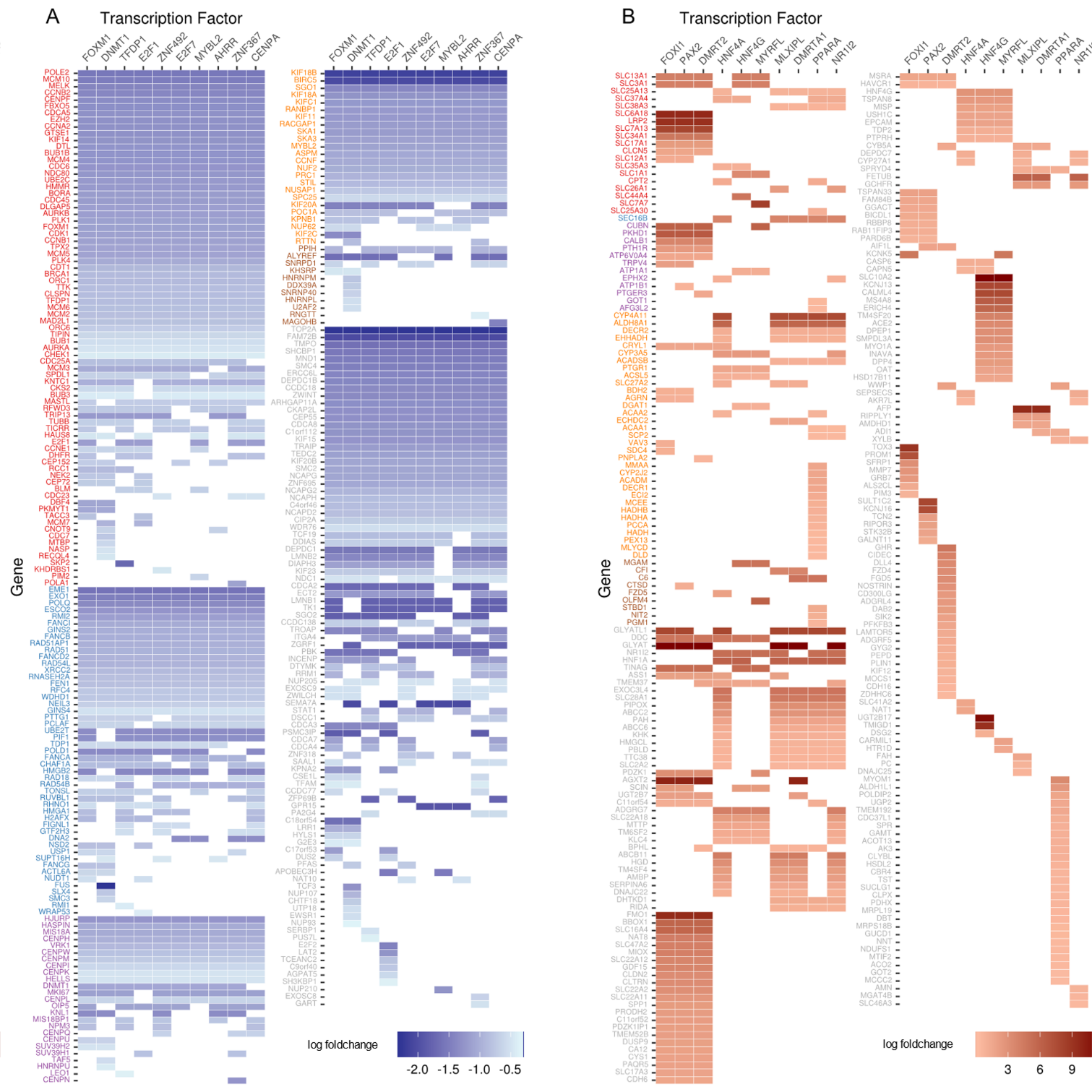


**a.**

**b.**
